# Frequencies and spectra of aflatoxin B_1_-induced mutations in liver genomes of NEIL1-deficient mice as revealed by duplex sequencing

**DOI:** 10.1093/narmme/ugae006

**Published:** 2024-05-17

**Authors:** Irina G Minko, Michael M Luzadder, Vladimir L Vartanian, Sean P M Rice, Megan M Nguyen, Monica Sanchez-Contreras, Phu Van, Scott R Kennedy, Amanda K McCullough, R Stephen Lloyd

**Affiliations:** Oregon Institute of Occupational Health Sciences, Oregon Health & Science University, Portland, OR, USA; Oregon Institute of Occupational Health Sciences, Oregon Health & Science University, Portland, OR, USA; Oregon Institute of Occupational Health Sciences, Oregon Health & Science University, Portland, OR, USA; Oregon Institute of Occupational Health Sciences, Oregon Health & Science University, Portland, OR, USA; School of Public Health, Oregon Health & Science University - Portland State University, Portland, OR, USA; Department of Laboratory Medicine and Pathology, University of Washington, Seattle, WA, USA; Department of Laboratory Medicine and Pathology, University of Washington, Seattle, WA, USA; TwinStrand Biosciences, Inc., Seattle, WA, USA; Department of Laboratory Medicine and Pathology, University of Washington, Seattle, WA, USA; Oregon Institute of Occupational Health Sciences, Oregon Health & Science University, Portland, OR, USA; Department of Molecular and Medical Genetics, Oregon Health & Science University, Portland, OR, USA; Oregon Institute of Occupational Health Sciences, Oregon Health & Science University, Portland, OR, USA; Department of Molecular and Medical Genetics, Oregon Health & Science University, Portland, OR, USA

## Abstract

Increased risk for the development of hepatocellular carcinoma (HCC) is driven by a number of etiological factors including hepatitis viral infection and dietary exposures to foods contaminated with aflatoxin-producing molds. Intracellular metabolic activation of aflatoxin B_1_ (AFB_1_) to a reactive epoxide generates highly mutagenic AFB_1_-Fapy-dG adducts. Previously, we demonstrated that repair of AFB_1_-Fapy-dG adducts can be initiated by the DNA glycosylase NEIL1 and that male *Neil1^−/−^* mice were significantly more susceptible to AFB_1_-induced HCC relative to wild-type mice. To investigate the mechanisms underlying this enhanced carcinogenesis, WT and *Neil1^−/−^* mice were challenged with a single, 4 mg/kg dose of AFB_1_ and frequencies and spectra of mutations were analyzed in liver DNAs 2.5 months post-injection using duplex sequencing. The analyses of DNAs from AFB_1_-challenged mice revealed highly elevated mutation frequencies in the nuclear genomes of both males and females, but not the mitochondrial genomes. In both WT and *Neil1^−/−^* mice, mutation spectra were highly similar to the AFB_1_-specific COSMIC signature SBS24. Relative to wild-type, the NEIL1 deficiency increased AFB_1_-induced mutagenesis with concomitant elevated HCCs in male *Neil1^−/−^* mice. Our data establish a critical role of NEIL1 in limiting AFB_1_-induced mutagenesis and ultimately carcinogenesis.

## Graphical Abstract

**Graphical Abstract:**
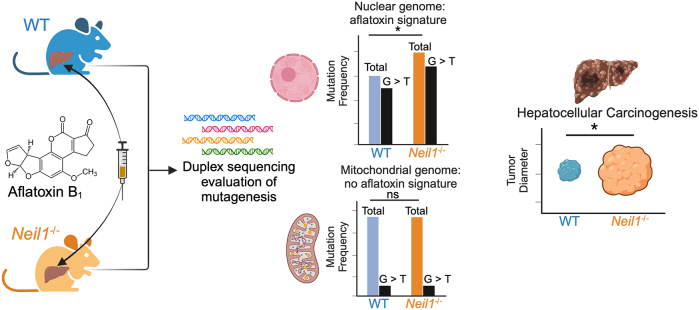

## Introduction

Hepatocellular carcinoma (HCC) is a leading cause of cancer death worldwide (1,2), with the highest occurrence in sub-Saharan Africa and Asia (1,3,4), in which HCCs are correlated with hepatitis B (HBV) infection and dietary aflatoxin B_1_ (AFB_1_) exposures (2,5–7). AFB_1_ is a mycotoxin produced by *Aspergillus flavus* (4,5). In regions with high dietary exposures to AFB_1_, such as Qidong County, China, there is a shift in the age of onset of HCC, in which males present in their 20s, peaking at ages 40–50, while in Beijing, where AFB_1_ exposures are minimal, early-onset HCC is not common (4,5).

Computational analyses of sequence variations in individual cancer genomes allowed the extraction of distinct mutational signatures (Catalog Of Somatic Mutations In Cancer (COSMIC) https://cancer.sanger.ac.uk/signatures/) associated with various internal biochemical deficiencies or exogenous challenges (8–10). These signatures differ by the spectra of mutations, the trinucleotide sequence contexts in which mutations preferentially occur, and the distribution of mutations throughout the genome with respect to replication timing, activity and directionality of transcription, and chromatin status. Using next-generation sequencing, analyses of HCCs from patients born in sub-Saharan Africa, with dietary exposures to AFB_1_ and infected with HBV, revealed ‘Signature Single Base Substitution (SBS) 24’ that is associated with AFB_1_ exposures (9). Subsequently, ‘Signature SBS24’ has been found in many additional liver cancers, including HCCs of patients from Qidong County, China (10–14), and its relation to AFB_1_ exposures was validated in several studies using cultured cells or animal models (12,14–17). These analyses consistently demonstrated the prevalence of C/G > A/T transversions as the major mutagenic event associated with AFB_1_ exposures and revealed that different sequence contexts were not equally susceptible to AFB_1_-induced mutagenesis, with SBS being clustered within a limited number of trinucleotide contexts. The reasons for such non-random mutation distribution are currently unclear.

Analyses of the pathways leading to aflatoxin-induced mutagenesis have revealed that following ingestion and uptake by the liver, AFB_1_ is primarily metabolized by cytochrome P450 3A4 to yield the AFB_1_-*exo*-8,9-epoxide (18–21). This electrophile alkylates N7-dG to form *trans*-8,9-dihydro-8-(N7-guanyl)-9-hydroxy aflatoxin B_1_ (22–24). This is a relatively short-lived species that either depurinates to release AFB_1_-guanine (22), or undergoes base-catalyzed opening of the imidazole ring to form *trans*-8,9-dihydro-8-(2,6-diamino-4-oxo-3,4-dihydropyrimid-5-yl-formamido)-9-hydroxy aflatoxin B_1_ (AFB_1_-Fapy-dG) (23,25–27). The AFB_1_-Fapy-dG lesion is persistent *in vivo* (25,28–32). The persistence, along with the known potential to cause high frequency base substitutions (80–90%), predominated by G > T transversions (33,34) implicate AFB_1_-Fapy-dG as a major contributor to AFB_1_-induced mutagenesis.

Two DNA repair pathways remove AFB_1_ adducts: nucleotide excision repair (NER) (29,35,36) and base excision repair (BER) (31,37–40). BER is initiated on AFB_1_-Fapy-dG adducts by hNEIL1 (human endonuclease VIII-like1) and the efficiency is modulated by sequence context, with ∼4-fold lower rates of excision when the adduct is positioned in a mutational hot spot (39). The biological significance for a role of the BER pathway was demonstrated by a >3-fold increase in HCCs in AFB_1_-treated *Neil1^−/−^* versus WT mice, along with greater accumulation of AFB_1_-Fapy-dG adducts in mouse liver DNAs (31).

Given the importance of NEIL1 in the maintenance of genomic integrity following aflatoxin exposures in a mouse model (31) and the possibility that pathogenic polymorphic variants of NEIL1 (37,40–42) may affect human HCC risk, the current study was designed to investigate whether the mutagenic frequencies and spectra, including distribution of mutations across trinucleotide sequences, are altered in NEIL1-deficient versus proficient mice. To address this question, we employed ultra-high accuracy Duplex Sequencing (DuplexSeq) (43–45). This DNA sequencing technology is designed to greatly reduce the artifacts of error-prone processes that occur during preparation and sequencing of DNA libraries and thus, it measures true sequence variations. Since mice only have a short optimal window for aflatoxin-induced carcinogenesis (30), newborn mice of both genotypes were exposed to a single AFB_1_ dose. This was followed by DuplexSeq analyses of the mutation profiles in selected regions of nuclear DNA (nDNA) and the entire mitochondrial DNA (mtDNA) genome 2.5 months post exposure to allow outgrowth for mutation fixation. Parallel cohorts of control and AFB_1_ exposed mice were followed for ∼15 months for the formation of tumors.

## Materials and methods

### Animals and tissue collection

All mice were maintained and handled according to the recommendations in the Guide for the Care and Use of Laboratory Animals of the National Institutes of Health. All procedures were approved by the Institutional Animal Care & Use Committee of the Oregon Health & Science University. The mice were housed under standard conditions with access to water and food *ad libitum*. The generation of *Neil1^−/−^* knockout mice was previously reported (46). The mating pairs of C57Bl/6J mice were obtained from Jackson Laboratories. The *Neil1^−/−^* mice were backcrossed > 15 generations on a C57Bl/6J background. For establishing the frequencies and spectra of spontaneous mutations, livers were harvested at 6 months of age (2 males and 2 females for analyses of nDNA or 3 males for analyses of mtDNA). For characterization of AFB_1_-induced mutations, 6-day old pups were injected with AFB_1_ at a dose of 4 mg/kg as previously described (31) and livers were harvested from 10 animals per group (5 males and 5 females) at ∼2.5 months of age. Following isolation, tissues were immediately flash-frozen, and stored at −80°C. Additional cohorts of AFB_1_-exposed WT and *Neil1^−/−^* mice were monitored for loss of weight and other health issues throughout the 15-month study. Any mouse visually experiencing health problems was euthanized and the morphology of tissues, including liver, was inspected. The remaining mice were euthanized at 15 months of age and similarly examined. Morphologically changed tissues were harvested, fixed, and analyzed as previously described (31).

### DNA isolation and fragmentation

DNA was isolated using DNeasy Blood and Tissue kit (Qiagen, catalog # 69504) with the following modifications. The homogenized liver tissues (21–25 mg) were incubated in 200 μl of the ATL/proteinase K solution at 50°C for 2 h, vortexed for 20 s every 15 min. Following digestion, samples were treated with 400 μg of RNase A (Qiagen, catalog # 19101), and incubated at room temperature for 5 min. All subsequent steps were performed according to the manufacturer's protocol. DNA concentrations were measured using either Quant-iT™ PicoGreen™ dsDNA Assay Kit or Qubit™ dsDNA HS Assay Kit (both from Invitrogen by ThermoFisher Scientific, catalog # Q33120 and Q33231, respectively). The quality of DNAs was tested on 1% agarose gels. For analyses of nuclear genomes, DNAs were diluted to 33 ng/μl in 10 mM Tris–HCl (pH 8.0), 1 mM EDTA and fragmented to a median size of ∼300 bp using a Covaris S220 ultrasonicator under the following conditions: 50 μl sample volume, 175 W peak incident power, 10% duty factor, 200 cycles per burst, 45 s treatment time, 7°C. Alternatively, DNA was enzymatically fragmented using the TwinStrand DNA fragmentation reagent (TwinStrand Biosciences, Seattle, WA). The size distributions of fragmented DNAs were analyzed on 2% agarose gels. mtDNA samples were prepared separately from nDNA libraries by fragmenting ∼100 ng of total DNA by sonication with a Covaris M220 ultrasonicator under the following conditions: 50 μl sample volume, 75 W peak incident power, 10% duty cycle, 200 cycles per burst, and 64 sec treatment time, and at ambient temperature.

### Preparation and sequencing of DNA libraries

To assess mutations in nuclear genomes, DNA libraries were prepared from 1 μg of pre-fragmented DNA using a TwinStrand DuplexSeq™ kit, supplemented with the Mutagenesis Panel (Mouse-50), v1.0. Briefly, the ends of fragmented DNA were repaired, dA-tailed, and ligated with adapters containing double-stranded unique molecular identifiers (UMIs). Common DNA base lesions and abasic sites were converted into DNA strand breaks to minimize the use of damaged DNA as template, and adapter-ligated DNA fragments were amplified by PCR in the presence of primers containing sample-specific dual indices. The target regions were captured by two rounds of hybridization with biotinylated bait oligodeoxynucleotides, each followed by PCR. DNA concentrations of the final libraries were measured using a Qubit™ dsDNA HS Assay Kit. The DNA size distributions were analyzed on 2% agarose gels. The libraries were sequenced on an IlluminaNovaSeq 6000 System (Illumina) by the OHSU Massively Parallel Sequencing Shared Resource, with a minimum of 200 million clusters per sample as a target (150-nt paired-end reads). Per sample sequence metrics are available in Supplementary Tables S1 and S2.

For analyses of mtDNA, sonicated samples were subjected to end-repair, dA-tailing, and ligation with double-stranded UMI adapters, using the NEB Ultra II library preparation kit (catalog # E7645) according to the manufacturer's protocol (New England Biolabs, Ipswitch, MA). Sequences for the double-stranded UMI adapters can be found in Sanchez-Contreras *et al.* (47,48). Targeted capture used the IDT xGen Lockdown protocol and probes specific for mouse mtDNA (Integrated DNA Technologies, Coralville, IA) following the manufacturer's instructions. The resulting libraries were indexed and then sequenced using 150-nt paired-end reads on an Illumina NovaSeq6000 with ∼20 × 10^6^ reads per sample. Per sample sequencing metrics are available in Supplementary Table S3.

### Data analyses

Raw sequencing data were de-multiplexed to obtain sample-specific FASTQ files. For analyses of nDNA, FASTQ files were uploaded onto the DNAnexus cloud platform (https://www.dnanexus.com) and processed using the TwinStrand DuplexSeq Mutagenesis application (version 3.21.0 and 3.20.1) developed and provided by TwinStrand Biosciences. This bioinformatic pipeline performed sequencing quality control, alignment against a reference genome and variant calling. It also provided information on sequencing quality metrics, calculated mutation frequencies and generated simple and trinucleotide mutation spectra. These data processing steps and the filtering parameters were used as previously described (49). The raw sequencing data obtained for mtDNA were processed using version 2.1.3 of our in-house bioinformatics pipeline (https://github.com/Kennedy-Lab-UW/Duplex-Seq-Pipeline), with the default consensus making parameters. A detailed description of the DuplexSeq pipeline has been previously reported (47,48).

The graphical data presentation, calculation of mean frequencies of mutations with standard errors, and evaluation of statistical significance of the differences by the two-tailed Student's *t*-test were performed using KaleidaGraph 4.1 software (Synergy Software, Reading, PA). Cosine similarity scores between the trinucleotide mutation spectra of samples and the mouse genome (assembly version mm10) SBS COSMIC signatures (v3.3) were determined using the R package Mutational Patterns (https://bioconductor.org/packages/release/bioc/html/MutationalPatterns.html) via the cos_sim_matrix function (50). Prior to comparison, the mm10 SBS COSMIC signatures were normalized by the trinucleotide abundance in the Mutagenesis Panel (Mouse-50) as performed (49) and described (51) in published studies. Trinucleotide spectra of nDNA and cosine similarity heatmaps were plotted using GraphPad Prism software (GraphPad Software, Boston, MA).

To evaluate the difference between diameters of tumors in WT and *Neil1^−/−^* mice, analyses were conducted using SPSS software version 29a. The mixed model that was applied to account for the intra-mouse correlation between tumors, revealed an intraclass correlation of 1, which precluded the application of such model. Thus, a two-step comparison test was performed. First, tumor sizes were compared in WT and *Neil1^−/−^* mice at the tumor level via independent samples *t*-tests. Next, because tumors were fundamentally not independent of one another, we also applied independent samples *t*-tests at the mouse level as a sensitivity test, using only one tumor per mouse for comparison. Significance was set at *P*< 0.05 for all tests. Due to the small sample size, Hedge's *g* effect size was also calculated.

## Results

### Experimental design

To evaluate potential roles of NEIL1 glycosylase in modulating the levels and/or spectra of somatic mutations in mouse liver DNA, both in unexposed animals and following AFB_1_ challenge, we employed ultra-high accuracy DuplexSeq (43–45). This method has been extensively used to study both spontaneous and induced mutagenesis (47–49,52–56). The background frequencies and spectra of mutations were determined in 6-month WT and *Neil1^−/−^* mice; for the analyses of nDNA, two males and two females were used per experimental group, while for the analyses of mtDNA, three males were used per experimental group. The AFB_1_-induced mutations were assessed in 2.5-month WT and *Neil1^−/−^* mice, using five males and five females per experimental group.

The high accuracy of DuplexSeq is accomplished by ligation of the initial DNA fragments with oligodeoxynucleotides, each containing two identifiers: a unique tag that is common for the two complementary strands in the same DNA molecule and an additional identifier that differentially marks top and bottom strand. DNA is amplified after ligation, and the target regions are captured using biotinylated probes. Following deep, next generation-based sequencing (NGS), the consensus sequences of the original DNA molecules are computed from multiple descendants of both strands.

The target regions in nDNA covered ∼48 kb and included both intergenic and genic autosomal sequences (20 total, Supplementary Table S4) that had been chosen to be representative of the whole genome with respect to GC-content and trinucleotide frequencies, except that CpG sites are underrepresented (Mutagenesis Panel (Mouse-50), v1.0, TwinStrand Biosciences). There are no indications that these regions are under significant positive or negative selection. The entirety of the 16.3 kb mouse mtDNA was separately targeted for capture with in-house designed biotinylated probes. The sequences are available in Sanchez-Contreras *et al.* (48).

### NGS and DuplexSeq mutagenesis assay metrics

For the analyses of nDNA, NGS provided data from ∼420–1300 million passing filter 150-nt reads per sample. The fraction of bases with ≥ Q30 ranged from ∼91 to ∼93%. Subsequent analyses using the TwinStrand pipeline revealed that ∼8–10% of the sequence data could be converted to duplexes, generating ∼1.7–2.0 billion informative ‘duplex’ bases per sample (Supplementary Tables S1 and S2). The mean on-target duplex depth ranged from ∼28 to ∼34.5 thousand. Reads were distributed ∼ uniformly across all targets in both unexposed (Supplementary Figure S1A) and AFB_1_-exposed animals (Supplementary Figure S1B). Since mtDNA exhibits mutation frequencies ∼100–1000× higher than nDNA (48,57), NGS provided data from ∼13–72 million 150-nt reads per sample, which resulted in ∼14–80 million ‘duplex’ bases per sample (Supplementary Table S3). The mean on-target duplex depth ranged from ∼867 to 4900.

### Spectra of mutations: analyses of individual samples

**Figure 1.**
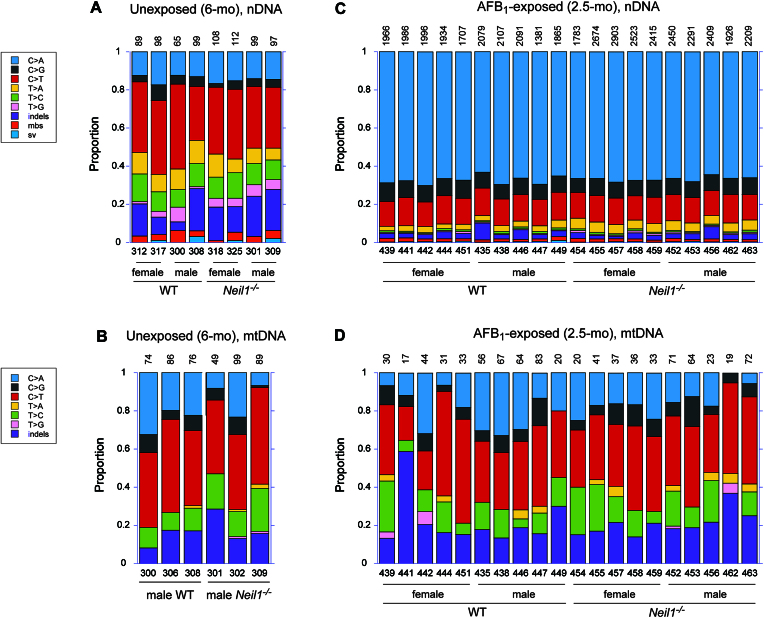
The proportion of different mutation types in individual samples in nDNA and mtDNA. (**A**) Spontaneous mutations in nDNA isolated from livers of 6-month mice. (**B**) Spontaneous mutations in mtDNA isolated from livers of 6-month mice. (**C**) Mutations in nDNA isolated from livers of AFB_1_-exposed 2.5-month mice. (**D**) Mutations in mtDNA isolated from livers of AFB_1_-exposed 2.5-month mice. Mutation types include SBS, insertions/deletions (indels), multiple base substitutions (mbs), and structural variants (sv). The numbers of mutations detected are shown on the top. The numbers below the bars refer to the mouse identification numbers. The corresponding numeric data are provided in Supplementary Tables S5–S8.

The TwinStrand and in-house developed DuplexSeq pipeline allowed for detection of various types of somatic mutations, including SBS, multiple base substitutions, insertion/deletions (indels), and structural variants. Sequence variants that were found more than once at the same position in the same sample were counted as a single mutation, as these were considered clonal in their origin. In nDNA obtained from unexposed animals, the number of unique mutations ranged from 65 to 112 (Figure 1A). The initial assessment of these samples revealed similar patterns in the proportions of different mutation types. These were dominated by SBS, with a prevalence of C/G > T/A transitions. In many of these samples, the proportion of indels was relatively high, accounting for up to ∼22% of total mutations. For mtDNA, the number of unique mutations ranged from 49 to 99. Similar to nDNA, SBS were the most common mutations (mean: 83.3%), with the spectra being dominated by C/G > T/A transitions (Figure 1B), a result consistent with previous studies of mouse mtDNA, both in terms of age-related frequency and mutational bias (48,58,59).

The number of mutations in nDNA isolated from AFB_1_-exposed animals was significantly higher, ranging from 1381 to 2523 unique variants per sample (Figure 1C). This set of samples also showed a low intra-individual variability regarding the mutation spectra. The most dominant type, accounting for ∼63–70% of all mutations, was C/G > A/T transversions, followed by ∼13–15% C/G > T/A transitions and ∼7–10% C/G > G/C transversions. Proportions of indels, multiple base substitutions, and structural variants were low; combined, these ranged from ∼3 to 6%, with the exception of two samples in which the occurrences of such sequence variations were ∼7 and 10% of the total. Overall, these patterns of mutations are highly consistent with the established mutagenic signature associated with AFB_1_ exposure (10–12,14–17). In contrast, strong bias towards C/G > A/T transversions was entirely absent in mtDNA from AFB_1_-exposed animals (Figure 1D).

### Frequencies and spectra of mutations: group comparison

**Figure 2.**
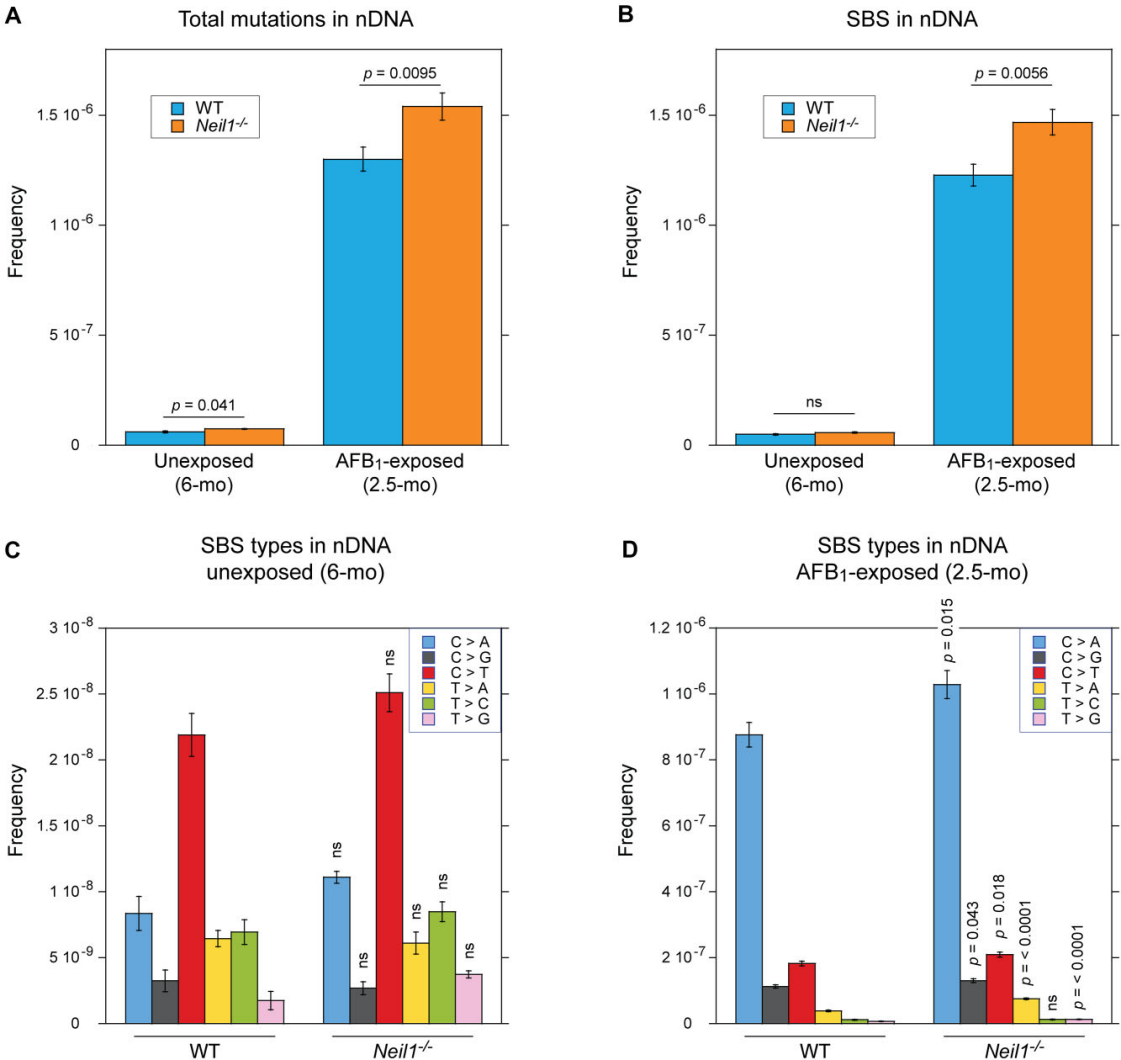
Frequencies and spectra of mutations in nDNA of WT and *Neil1^−/−^* mice. (**A**) Frequencies of total mutations in nDNA isolated from livers of 6-month unexposed and 2.5-month AFB_1_-exposed mice. (**B**) Frequencies of SBS in nDNA isolated from livers of 6-month unexposed and 2.5-month AFB_1_-exposed mice. (**C**) Types of SBS in nDNA isolated from livers of 6-month unexposed mice. (**D**) Types of SBS in nDNA isolated from livers of 2.5-month AFB_1_-exposed mice. Group sizes: *n* = 4 per genotype for unexposed mice and *n* = 10 per genotype for AFB_1_-exposed mice. Mean frequencies are plotted. Error bars represent standard errors and *P*-values were calculated via two-tailed Student's *t*-test for pairwise comparisons between WT and *Neil1^−/−^* for all mutations (A), SBS (B), or SBS types (C and D). The corresponding numeric data are provided in Supplementary Tables S9–S12.

Mutation frequencies were calculated as a ratio of the identified unique mutated sites to the total number of the target bases that could be unambiguously called. Figure 2 shows the nDNA mean frequencies of all detected mutations (Figure 2A) and SBS (Figure 2B) for each experimental group, with both being significantly higher in AFB_1_-exposed animals. Relative to the background levels, overall mutagenesis was elevated ∼22-fold in WT mice ((5.97 ± 0.48) × 10^−8^ versus (1.30 ± 0.06) × 10^−6^) and ∼21-fold in *Neil1^−/−^* mice ((7.35 ± 0.23) × 10^−8^ versus (1.54 ± 0.06) × 10^−6^); SBS were elevated ∼25-fold in WT mice ((4.86 ± 0.37) × 10^−8^ versus (1.23 ± 0.05) × 10^−6^) and ∼26-fold in *Neil1^−/−^* mice ((5.72 ± 0.28) × 10^−8^ versus (1.47 ± 0.06) × 10^−6^). This was despite the older age of unexposed animals (6 months versus 2.5 months). The increase in overall SBS frequency in 2.5-month AFB_1_-exposed mice relative to 6-month unexposed mice was reflected in increased frequencies across all mutation subtypes with C/G > A/T transversions being the most common in both experimental groups. Regarding the comparison of the two genotypes, frequencies of spontaneous somatic mutations were higher in *Neil1^−/−^* mice (by ∼23% if all types of mutations were counted and ∼18% if only SBS were counted), with the difference in frequencies of total sequence variations being statistically significant (Figure 2A). NEIL1 deficiency also resulted in increased mutational levels in AFB_1_-exposed animals; relative to WT, frequencies of total mutations and SBS were elevated by ∼18% and 20%, respectively (Figure 2B). When DNAs from exposed mice were grouped by sex, comparable genotype-dependent differences were observed. Consistent with results of the earlier study (30), the comparison of female and male groups within the same genetic background showed no significant differences in AFB_1_-induced mutagenesis (Supplementary Figure S2).

Analyses for specific types of base substitutions in nDNA from unexposed animals confirmed the initial observation (Figure 1) that C/G > T/A transitions are the most prevalent spontaneous base substitutions in liver nDNA at 6 months of age (Figure 2C). Although these, as well as C/G > A/T transversions, T/A > C/G transitions, and T/A > G/C transversions were elevated in *Neil1^−/−^* mutants, the differences were statistically insignificant. In AFB_1_-exposed animals, almost all types of base substitutions, except for T/A > C/G transitions, accumulated at higher frequencies in the absence of NEIL1 (Figure 2D). These included signature C/G > A/T transversions that occurred at ∼17% increased frequency. Comparable, ∼15% increases were also observed for C/G > G/C transversions and C/G > T/A transitions. Although frequencies of base substitutions at T/A sites were relatively low in both experimental groups, ∼2-fold higher occurrences of T/A > A/T and T/A > G/C transversions were detected in NEIL1-deficient mice.

**Figure 3.**
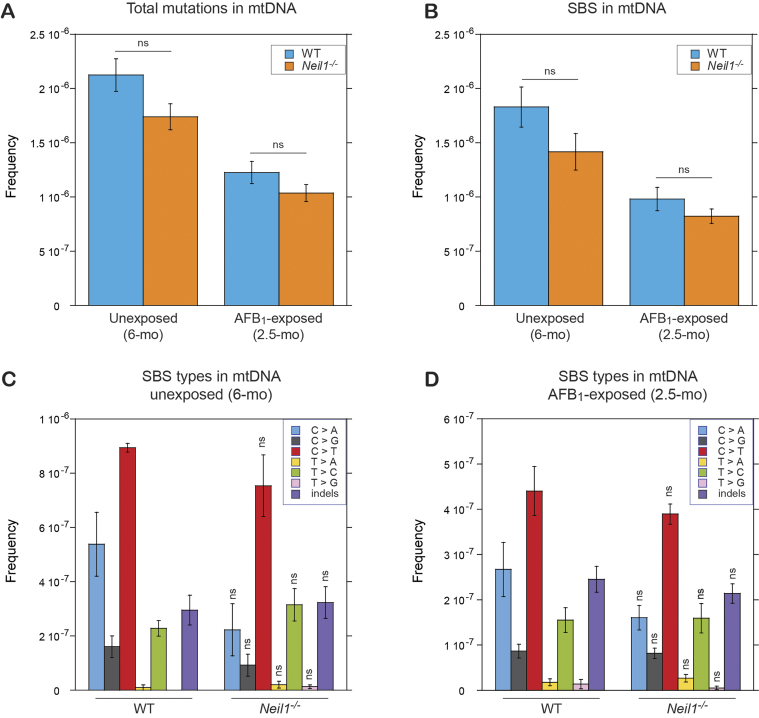
Frequencies and spectra of mutations in mtDNA of WT and *Neil1^−/−^* mice. (**A**) Frequencies of total mutations in mtDNA isolated from livers of 6-month unexposed and 2.5-month AFB_1_-exposed mice. (**B**) Frequencies of SBS in mtDNA isolated from livers of 6-month unexposed and 2.5-month AFB_1_-exposed mice. (**C**) Types of SBS in mtDNA isolated from livers of 6-month unexposed mice. (**D**) Types of SBS in mtDNA isolated from livers of 2.5-month AFB_1_-exposed mice. Group sizes: *n* = 3 per genotype for unexposed mice and *n* = 10 per genotype for AFB_1_-exposed mice. Mean frequencies are plotted. Error bars represent standard errors and significance was calculated via two-tailed Student's *t*-test. The corresponding numeric data are provided in Supplementary Tables S13–S16.

In contrast to the results in nDNA, mtDNA exhibited no increase in total mutations or SBS (Figure 3) in AFB_1_-exposed animals. In fact, mutation frequencies were lower in this group relative to unexposed animals, likely because of the age difference. Additionally, there were no differences observed between WT and *Neil1^−/−^* samples, either with or without AFB_1_ exposure. Given the lack of effect of NEIL1 knockout or AFB_1_ exposure on mtDNA mutagenesis, we focused the rest of our analyses on the nDNA.

### Trinucleotide mutation spectra and comparison to COSMIC signatures

**Figure 4.**
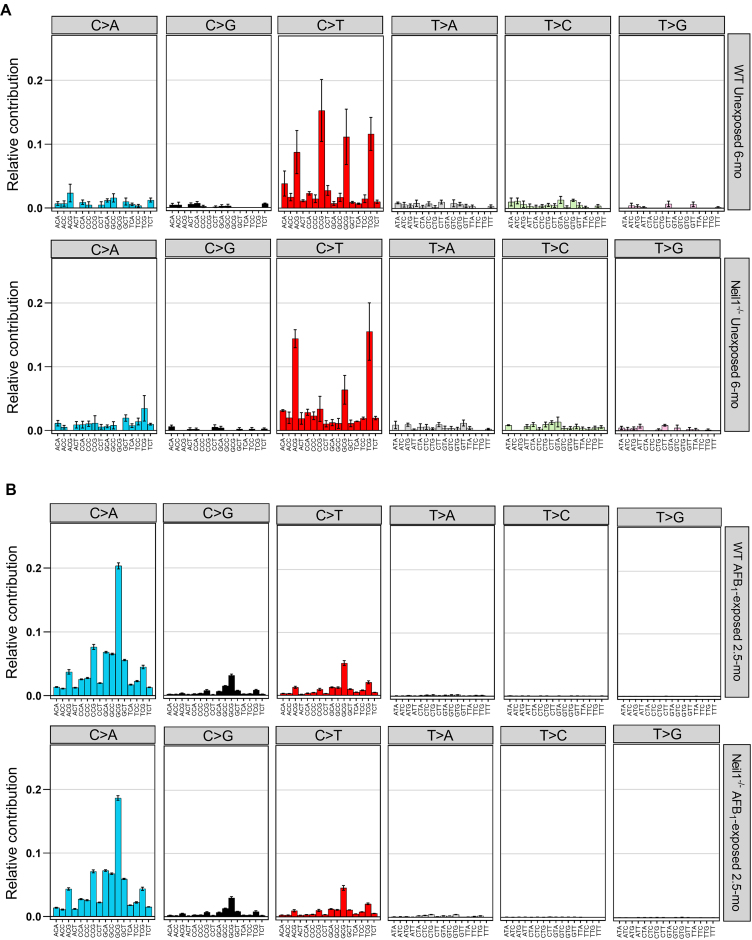
The trinucleotide mutation spectra in nDNA of (**A**) unexposed 6-month and (**B**) AFB_1_-exposed 2.5-month WT and *Neil1^−/−^* mice. Data are presented as mean proportions and error bars represent standard error. Group sizes: *n* = 4 per genotype for unexposed mice and *n* = 10 per genotype for AFB_1_-exposed mice. The corresponding numeric data are provided in Supplementary Tables S17 and S18.

The influence of local sequence context on mutation frequency in both unexposed and AFB_1_-exposed WT and *Neil1^−/−^* mice was evaluated by considering the 3′ and 5′ nucleotides flanking each mutation. Trinucleotide mutation frequencies in nDNA were calculated as the ratio of mutations in a trinucleotide context to the total number of occurrences of that trinucleotide context. These analyses revealed a different distribution of SBS, most notably C/G > T/A transitions, in unexposed *Neil1^−/−^* mice relative to unexposed WT mice (Figure 4A). To validate this observation, we compared the genotypes with respect to the frequencies of mutations in each trinucleotide sequence. Student's *t*-tests showed significantly decreased frequencies of CCG > CTG (CGG > CAG) ((8.42 ± 4.86) × 10^−8^ versus (3.52 ± 0.76) × 10^−7^; *P* = 0.025) and increased frequencies of TCA > TTA (TGA > TAA) ((3.87 ± 0.45) × 10^−8^ versus (1.66 ± 0.04) × 10^−8^; *P* = 0.0028) and TCT > TTT (AGA > AAA) ((5.23 ± 0.74) × 10^−8^ versus (2.51 ± 0.65) × 10^−8^; *P* = 0.032) in *Neil1^−/−^* mice; statistical analyses are given as standard errors. While statistically significant differences also existed in the frequency of mutations in the trinucleotide mutation spectra between AFB_1_-exposed WT and *Neil1^−/−^* mice, these differences are likely not biologically relevant as the overall trinucleotide mutation spectra are highly similar between AFB_1_-exposed WT and *Neil1*^−/−^ mice (cosine similarity: AFB_1_-exposed WT versus *Neil1*^−/−^ = 0.98) (Figure 4B). This may suggest that the significant increase in overall SBS mutation frequency observed in nDNA of AFB_1_-exposed *Neil1^−/−^* mice relative to AFB_1_-exposed WT mice is due to an increase in mutations across all mutation subtypes and trinucleotide contexts, rather than enrichment or depletion of a particular sequence context or mutation subtype.

**Figure 5.**
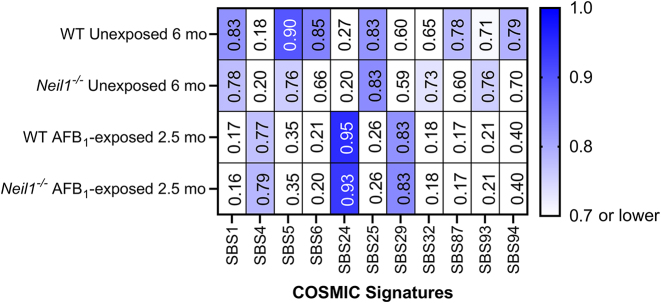
Heatmap of cosine similarity scores between trinucleotide mutational spectra of unexposed 6-month and AFB_1_-exposed 2.5-month WT and *Neil1^−/−^* mice and mm10 SBS COSMIC signatures. Signatures with cosine similarity below 0.7 in all samples were not plotted. The etiology proposed in the COSMIC database for these signatures are as follows: SBS1 - spontaneous deamination of 5′ methylcytosine (clock-like); SBS4 – tobacco smoke; SBS5 – unknown with clock-like manifestation; SBS6 – defective DNA mismatch repair; SBS24 – aflatoxin exposure; SBS25 – chemotherapy; SBS29 – tobacco chewing; SBS32 – azathioprine treatment; SBS87 – thiopurine chemotherapy treatment; SBS93 and SBS94 – unknown (https://cancer.sanger.ac.uk/signatures/).

The trinucleotide mutation spectra in nDNA of AFB_1_-exposed 2.5-month and unexposed 6-month WT and *Neil1^−/−^* mice were compared to the mm10 SBS COSMIC signature database (v 3.3) and cosine similarity scores were determined. Prior to comparison, the mm10 SBS COSMIC signatures were normalized relative to the abundance of trinucleotide contexts in the TwinStrand Biosciences Mutagenesis Panel (Mouse-50). These analyses revealed that the trinucleotide mutation spectra of unexposed 6-month WT mice and *Neil1^−/−^* mice were both highly similar to the clock-like signatures (8,60) SBS1 (cosine similarity: unexposed WT = 0.83, unexposed *Neil1^−/−^* = 0.78) and SBS5 (cosine similarity: unexposed WT = 0.90, unexposed *Neil1^−/−^* = 0.76) (Figure 5). The mutation spectra of AFB_1_-exposed 2.5-month WT and *Neil1^−/−^* mice were most similar to SBS24 associated with aflatoxin exposure (cosine similarity: AFB_1_-exposed WT = 0.95, AFB_1_-exposed *Neil1^−/−^* = 0.93) (Figure 5) (10–12,14).

### Distribution of mutations across genomic targets

**Figure 6.**
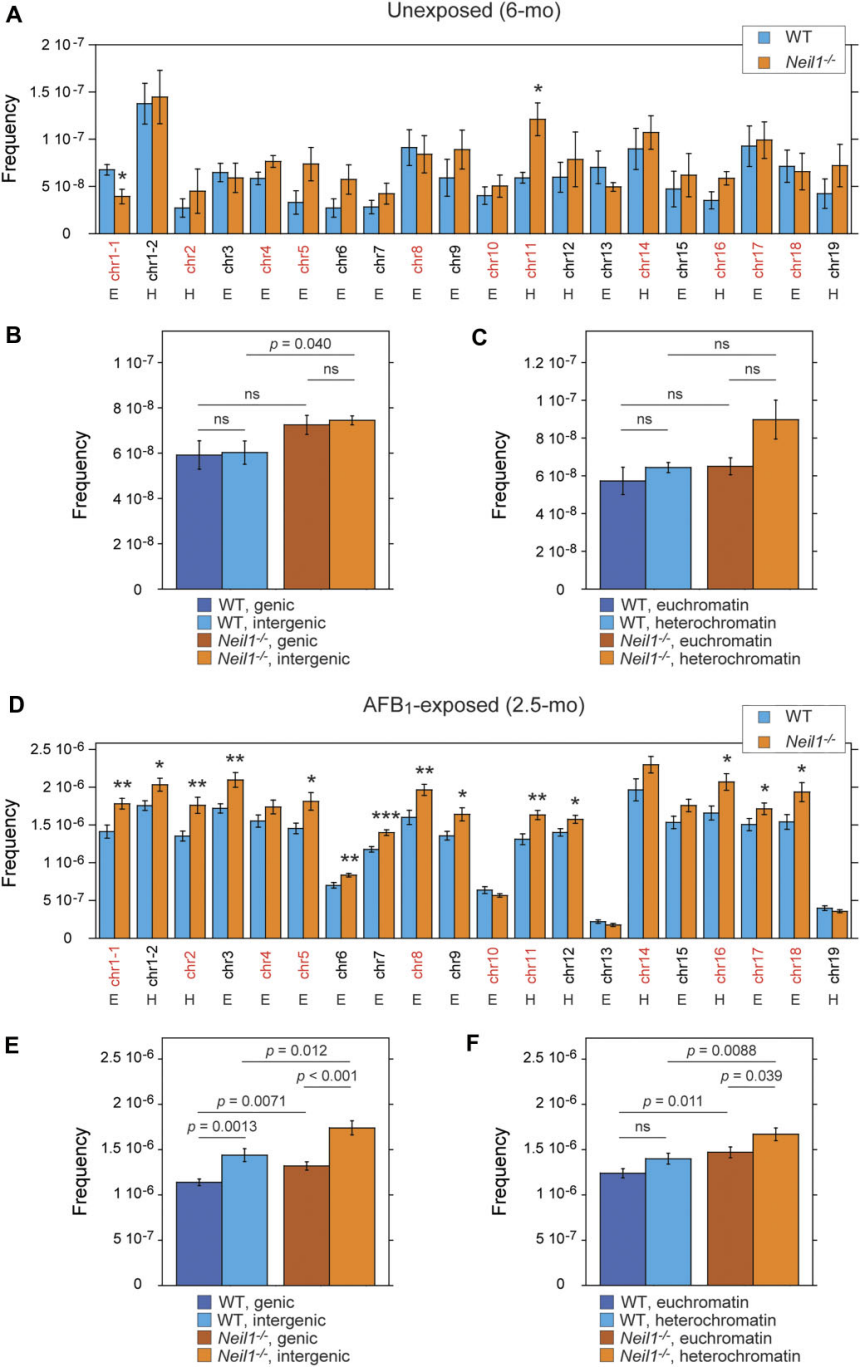
Distribution of mutations across genomic targets. Mean frequencies of spontaneous (**A–C**) and AFB_1_-induced (**D–F**) mutations. The frequencies were calculated for each individual target (A, D) and for targets grouped by location in either genic or intergenic region (B, E) or by chromatin status (C, F). Error bars represent standard errors. Intergenic targets are in red and genic targets are in black. The targets present in either heterochromatin (H) or euchromatin (E) locations. Group sizes: n = 4 per genotype for unexposed mice and *n* = 10 per genotype for AFB_1_-exposed mice. Significance was calculated using two-tailed Student's *t*-test for each target for comparison of WT and *Neil1^−/−^* genotypes: *P ≤ 0.05; **P ≤ 0.01; ***P ≤ 0.001. The corresponding numeric data are provided in Supplementary Tables S19–S21.

The TwinStrand Mutagenesis Panel (Mouse-50) is designed to target 20 regions that are ∼2.4 kb each on 19 out of 20 autosomes in the mouse nuclear genome (2 targets on chromosome (chr) 1, with no target on chr20) (Supplementary Table S4). These analyses revealed uneven distribution of mutations across these targets in both unexposed and AFB_1_-exposed animals (Figure 6). The frequencies of spontaneous mutations in the least and most affected targets differed by ∼5-fold in WT (chr2 and chr6 versus target 2 on chr1) and ∼4-fold in *Neil1^−/−^* mice (target 1 versus target 2 on chr1) (Figure 6A). Although the distribution patterns were generally similar between the two genotypes, target 1 on chr1 appeared to be more prone to spontaneous mutagenesis in WT mice relative to *Neil1^−/−^*, while the target on chr11 showed an opposite relationship. In both genotypes, no differences in frequencies of spontaneous mutations were found between the intergenic and genic chromosome regions (Figure 6B). The frequencies of spontaneous mutations were elevated in the heterochromatin versus euchromatin locations in both WT and *Neil1^−/−^* mice, but statistical analyses did not reveal significance (Figure 6C). Relative to unexposed animals, the difference between the least and most affected targets in AFB_1_-exposed animals was higher, ∼9-fold in WT and ∼13.5-fold in *Neil1^−/−^* (chr13 versus chr14 in both genotypes) (Figure 6D). The distribution patterns were remarkably similar between the genotypes. The targets on chr6, chr10, chr19 and chr13 accumulated significantly fewer mutations relative to other targets. Frequencies of AFB_1_-induced mutations in the genic targets were ∼30% lower in both genotypes (Figure 6E). Similar to spontaneous mutations, AFB_1_-induced mutations were relatively more abundant in the heterochromatin locations; in the *Neil1^−/−^* cohort, the difference was statistically significant (Figure 6F). Consistent with analyses of the total mutation frequencies (Figure 2A), most targets in nDNA of *Neil1^−/−^* mice were more prone to AFB_1_-induced mutagenesis relative to the corresponding targets in WT mice (Figure 6D).

### Hepatocellular carcinogenesis in AFB_1_-treated *Neil1^−/−^* mice

**Figure 7.**
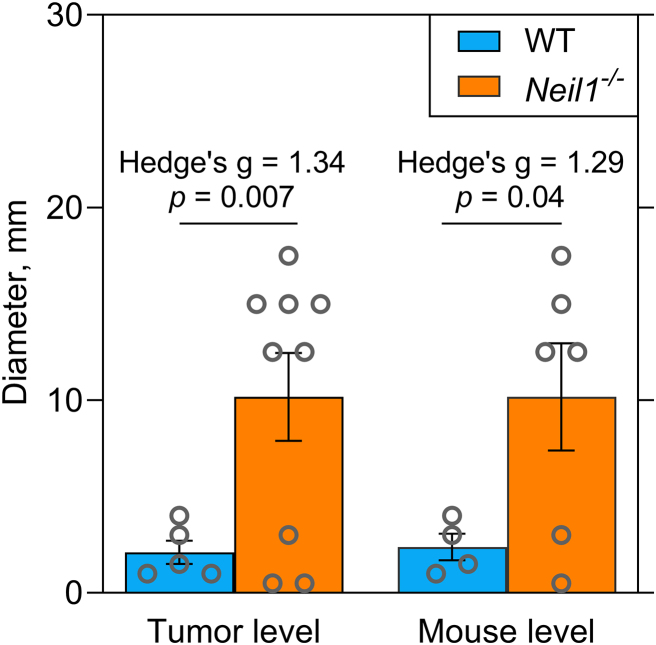
The size of liver tumors observed in AFB_1_-exposed WT and *Neil1^−/−^* male mice. The average diameters with standard errors are shown. Significance was calculated using independent sample *t*-tests and effect sizes were calculated using Hedge's g. Statistical comparisons were performed at both the tumor level and mouse level. At the tumor level, group sizes are *n* = 5 for WT and *n* = 9 for *Neil1^−/−^*. At the mouse level, group sizes are *n* = 4 for WT and *n* = 6 for *Neil1^−/−^*. The corresponding numeric data are provided in Supplementary Table S22.

Previously, we have reported that *Neil1^−/−^* mice developed greater tumor burdens relative to WT control mice following single injections of either 1.0 or 7.5 mg/kg doses of AFB_1_, with greater incidences in males versus females (31). In this current investigation, we also assessed tumor formation in both genotypes at the same dose as used in the DuplexSeq studies. Newborn WT and *Neil1^−/−^* mice were injected with AFB_1_ in DMSO, but at a dose of 4.0 mg/kg or a no-dose control. Injected mice were immediately returned to their home cage, weaned at 3–4 weeks, and housed until 15 months. Following euthanasia, livers were collected, and the frequencies and sizes of tumors analyzed with tissues both flash-frozen or formalin fixed. For WT mice, 9 males and 9 females were analyzed, with 5 mice of each gender challenged with AFB_1_. For *Neil1^−/−^* mice, 14 males and 15 females were carried throughout the study, with 6 males and 5 females injected with AFB_1_. All control, non-AFB_1_ injected mice were tumor-free at the termination of the study. Three out of ten AFB_1_-challenged females developed tumors, with one and two tumors observed in *Neil1^−/−^* and WT mice, respectively (Supplementary Table S22). AFB_1_-exposed male mice showed large increases in HCC formation, with tumor diameters measured at the time of euthanasia. Relative to WT animals, HCCs in *Neil1^−/−^* mice were not only more prevalent, but also significantly larger, with the average tumor diameter being ∼5-fold greater (Figure 7 and Supplementary Table S22). Overall, these data align well with prior investigations (31,61) and emphasize that there are strong, sex-specific differences in factors that drive carcinogenesis, even though there were not sex-specific differences in mutation frequencies and spectra in the 2.5-month mice (Supplementary Figure S2).

## Discussion

### Complexities of gene-environmental factors affecting mutational signatures—aflatoxin exposure as a model system

Disease manifestations of environmental mutagen exposures are most readily evidenced by increases in tissue-specific carcinogenesis. The final carcinogenesis outcome is modulated by complex processes including, but not limited to (i) the route, concentration, duration, and timing of exposure, (ii) whether bioactivation is a prerequisite of DNA adduct formation, (iii) the rates of detoxication and clearance pathways, (iv) the presence of concomitant genotoxicant challenges that can arise from either endogenous or exogenous sources, (v) repair and replication status of exposed cells, (vi) activation or evasion of cell-death pathways, and (vii) sex-specific modulators of transformation. However, following exposure and if necessary, bioactivation, there are further complicating factors that can modulate mutational outcomes, including intracellular distribution of the mutagenic agent, its base- and sequence-context specific binding, the degree of chromatin condensation and accessibility, the phase of the cell cycle, and expression levels of enzymes and accessory proteins involved in DNA damage repair and tolerance. When a mutation spectrum is established, that signature represents the amalgamation of these factors and modifiers of genetic instability. Toward developing a comprehensive understanding of the relative balance and importance of factors that will ultimately influence both the mutagenic and carcinogenic consequences of exposure to a genotoxic agent, we have chosen AFB_1_-induced mutagenesis and carcinogenesis as a model system. The advantages of choosing AFB_1_ are numerous given its role in HCC induction in humans and animal species. These include the combined knowledge of organ-specific bioactivation and detoxication pathways, the structure and stability of AFB_1_ DNA adducts, the effects that these adducts have on the fidelity of DNA replication, and the roles of two DNA repair pathways, NER and BER, that actively reduce adduct burden, contributing to the establishment of a distinctive mutation signature in cancers and *in vivo* models.

### The role of error-prone replication past AFB_1_ adducts in conferring final mutation frequencies and signatures

The ultimate driver of mutagenesis is error-prone translesion synthesis that is necessitated by stalled replication complexes at sites of unrepaired DNA adducts. We previously showed that yeast Rev3, a catalytic subunit of polymerase (pol) ζ, incorporates dA opposite AFB_1_-Fapy-dG and extends from the AFB_1_-Fapy-dG:dA mismatch (33). These data are consistent with the AFB_1_ mutagenic spectrum, showing G > T transversions (12,14–17,30,62). Further support for a major role for pol ζ in error-prone translesion synthesis past AFB_1_-Fapy-dG adducts was shown using mouse embryo fibroblast (MEF) cells that were deficient in pol ζ (*Rev3L^−/−^*). When these cells were exposed to AFB_1_, survival was reduced relative to *Rev3L^+/−^* cells or *Rev3L^−/−^* cells complemented with WT human REV3L (63). After exposure, cell-cycle progression of *Rev3L^−/−^* cells was arrested in late S/G2 phase and associated with replication-dependent formation of γ-H2AX foci, micronuclei, and chromatid breaks and radials. In addition, pol η and pol κ have the potential to contribute to mutagenic bypass of both the initial N7-dG and Fapy lesions (33,64). Consistent with an extremely error-prone bypass of AFB_1_-induced adducts observed *in vitro*, the biological consequences of blocked replication and subsequent translesion synthesis past these lesions are high frequency SBS, predominantly G > T transversions (33,34,64). Although these spectra strongly correlate with the mutagenic outcome of AFB_1_ exposure (12,14–17,30,62), they have been shown to be only minimally modulated by sequence context. Thus, mutational hot spots in the AFB_1_ signature cannot be explained by sequence-dependent differences in fidelity of replication past AFB_1_-Fapy-dG.

### The role of BER in modulating mutagenic and carcinogenic consequences of AFB_1_ exposure

Since there is a strong correlation between deficiencies in DNA repair and elevated risks of cancer development, as illustrated with diseases such as xeroderma pigmentosum (65) and colon cancer (66), investigations of the role of DNA repair deficiencies in cancer risk assessment are well justified. In regard to aflatoxin-driven HCC, although a role for NER has been well documented for decades (29,35,36), the observation that AFB_1_-FapyGua adducts were excised by NEIL1 was unexpected, given that this adduct is a sterically large base modification and its aromatic portion is intercalated on the 5′-face of the damaged guanine, providing thermal stability to duplex DNA (39,67,68). However, the biological manifestations of aflatoxin exposure in combination with the loss of NEIL1 in mice resulted in a multifold increase in HCC formation (31). To investigate the relative contributions of the DNA repair pathways to the mutation spectra and carcinogenesis, we developed murine strains in which the genotypes only varied by the expression of *Neil1*, while controlling for the concentration, timing, and route of the administration of aflatoxin, the sex of the exposed mice, the timing of tissue harvesting and analyses, and the methodologies through which these comparative analyses would be performed. Thus, the experimental design of this study was tailored to probe whether a NEIL1 deficiency correlated with changes in mutation frequencies or spectra.

Sequence analyses of nDNA from unexposed mice revealed a predominance of SBS, accounting for ∼80% of all mutations and a prevalence of C/G > T/A transitions. The occurrence of these transitions, at least partially, can be attributed to spontaneous deamination of 5-methylcytosines; the corresponding clock-like signature (SBS1) was present in the trinucleotide mutation spectra in nDNA of both WT and *Neil1^−/−^* mice. Comparative analyses of NEIL1-proficient versus deficient animals showed differences in frequencies of spontaneous mutations as well as their distributions across trinucleotide sequences and genomic targets. However, considering the small numbers of animals per experimental group (*N* = 4) and low mutation density in each sample, further studies are needed to confirm the essential role of NEIL1 in modulating spontaneous mutagenesis in nDNA in liver tissues.

In contrast to the mutation frequencies and spectra measured in nDNA from liver tissue in 6-month unexposed mice, nDNA isolated 2.5 months post exposure to a single 4.0 mg/kg dose of AFB_1_ showed both an increase in overall mutations and a different spectrum. In both the WT and NEIL1-deficient mice, the mutation spectra were dominated by C/G > A/T transversions (∼63–70%), followed by C/G > T/A transitions (∼13–15%) and C/G > G/C transversions (∼7–10%). The specificity of the mutations and sequence context-dependent distribution were highly consistent with SBS24. An additional similarity with SBS24 was the genic versus intergenic asymmetry, with the genic chromosome regions being better protected against AFB_1_-induced mutagenesis. Three of the four least affected targets (chr6, chr13, and chr19) are in the genic regions (Supplementary Table S4). Comparison of our results with the published studies in which the same TwinStrand mouse mutagenesis panel was used, revealed both similarities and differences. Analogous to results of the current investigation, targets on chr13 and chr19 accumulated relatively low levels of mutations in bone marrow of MutaMouse following exposure to either benzo(a)pyrene (49) or procarbazine hydrochloride (56). The intergenic target on chr14 was heavily loaded with mutations in all three cases. In contrast, one of the least affected targets in bone marrow, chr3, showed a high level of AFB_1_-induced mutations in liver nDNA. Collectively, these data demonstrate uneven protection of different regions in nDNA against induced mutagenesis that could be tissue- and/or mutagen-specific or depend on the age of animals at the time of exposure.

Our data revealed that a potential role of NEIL1-mediated suppression of AFB_1_-induced carcinogenesis in C57Bl6 mice is consistent with reduced mutagenesis, such that in the absence of NEIL1, overall mutation frequencies were elevated by ∼20%. Based on biochemical data showing an ∼4-fold, T_m_-dependent difference in the efficiency of NEIL1-catalyzed excision of AFB_1_-Fapy-dG (39), we had anticipated a modulation of the trinucleotide AFB_1_ mutation spectrum in *Neil1^−/−^* mice. However, the mutation types and distributions were largely unchanged between the WT and NEIL1-deficient mice. We hypothesize that thermal stability of DNA at the lesion site may be a key factor that influences sequence-dependent AFB_1_-induced mutagenesis. AFB_1_-Fapy-dG in a more thermally stable environment is likely to be more refractory not only to NEIL1-initated repair, but also NER. Such differential thermal stability could also direct the choice between error-prone replication bypass versus error-free damage avoidance pathways.

The unexpected result was an ∼2-fold increase in frequencies of A/T > T/A and A/T > C/G transversions in the NEIL1-deficient background. This observation suggests that following AFB_1_ exposure, either deoxyadenosine (dA) or deoxythymidine undergoes modification to form a DNA lesion that is a substrate for NEIL1. We speculate that this lesion could be an AFB_1_-substituted Fapy-dA adduct based on the following: replication of vectors treated with activated AFB_1_ results in mutations not only at C/G, but also at T/A sites (69); modification of DNA by AFB_1_ creates alkali-labile lesions at adenines, in addition to guanines (70); the N7 position of adenine is susceptible to alkylation (71), which by analogy with guanine, may lead to imidazole ring-opening to form substituted Fapy-dA adduct; and unsubstituted Fapy-dA is a substrate for NEIL1 (72).

Although overall mutation frequencies were elevated in NEIL1-deficient mice relative to wild-type mice, this increase was modest relative to the multifold increases in the tumor diameters. Insights into the origins of these differences may reside in the choice of DNA targets utilized in establishing the mutation frequencies and spectra. In this regard, it should be noted that the design of DNA sequencing panels by TwinStrand duplex sequencing technology avoided influences of either positive or negative selection during the challenge and subsequent fixation of mutations (54,55). Since this design specifically avoids confounding drivers that may strongly influence cell death or transformation, thereby influencing final tumor growth, assessment of mutagenesis in tumor promoter and tumor suppressor genes may provide insights into the increased tumorigenesis in NEIL1-deficient mice. To gain a better understanding of the impact of target choice for duplex sequencing, future directions will include systematic analyses of mutagenesis in genes associated with transformation and further complemented with DNA sequence analyses of mutations associated with aflatoxin-induced HCC in NEIL1-deficient and proficient strains.

In addition to insights that may be gained from expanding gene-specific mutational patterns, we hypothesize roles for NEIL1 beyond the initiation of BER as potential factors in hepatocarcinogenesis. These may include its non-catalytic functions in stabilization of replication forks (73) and active DNA demethylation (74). Recent investigations have also demonstrated that NEIL1 acts as a transcription factor in the initiation of colorectal cancers through regulation of the expression of COL17A1 (75). This study demonstrated that upregulation of NEIL1 enhances colorectal cancer initiation through the formation of an RNA polymerase II transcriptional complex with SATB2 and cMyc, which drives COL17A1 expression leading to cancer-associated immunosuppression.

### Differential mutation burden in nuclear versus mitochondrial DNAs

The mitochondrial DNA genome consists of an ∼16 kb circular molecule that encodes critical components of the electron transport chain in which mutations can cause severe maternally-inherited diseases, as well as common diseases of aging (76,77). Recent studies have tied somatically-acquired mtDNA mutations to carcinogenesis (78,79). Indeed, somatic mtDNA mutations and a reduction of copy number are thought to be common events in HCC, suggesting that a mitochondrial dysfunction-induced cascade may play a part in HCC progression (80–82).

Given the increased burden for elevated base damages within the mitochondria, especially those arising from the generation of reactive oxygen species, mitochondrial BER can be initiated by multiple DNA glycosylases including UNG1, MUTYH, OGG1, NEIL1, and NTH1 (83,84). The biological significance of mitochondrial BER in limiting the development of obesity and metabolic syndrome has been demonstrated through analyses of C57Bl6 mice deficient in *Ogg1* (85–87). *In vivo* correction of Ogg1-initiated mtDNA repair by transgenic expression of a mitochondria-targeted human OGG1 resulted in resistance to obesity and metabolic syndrome (85,88,89). Although metabolic syndrome has been reported in *Neil1^−/−^* mice, with an associated decrease in mtDNA integrity (46,90), correction of mitochondria-specific repair by transgenic NEIL1 expression has not been reported. However, in the male *Neil1^−/−^* mice > 9 months of age, both qPCR and Southern blot data indicated increased levels of damage and deletions in mtDNA (46), thus potentially linking loss of mtDNA integrity with metabolic syndrome in that model. This loss of NEIL1-initiated mitochondrial repair is an additional potential contributor to the carcinogenic phenotype in NEIL1-deficient mice.

While the repertoire of repair mechanisms in mtDNA is limited, mitochondria are subjected to autophagic quality control mechanisms (82,91). These mechanisms have been hypothesized to remove dysfunctional mitochondria that contain either mutated or damaged mtDNA. Numerous other investigations have suggested that mtDNA mutations are under some level of negative selection, but the specific mechanism(s) involved are not known (48,92,93).

Data presented herein demonstrate that AFB_1_ exposures, which result in greater than an order of magnitude increase in nDNA mutations, generated no additional mutational burden in mtDNA. A potential explanation for these results could include low levels of AFB_1_ DNA adduct formation in mtDNA and an inability of DNA polymerase γ to replicate past these lesions. Concerning AFB_1_ DNA adduct formation in mammals, prior studies in Sprague-Dawley rats demonstrated that *in vivo* exposures to comparable doses of AFB_1_ (6.0 mg/kg), generated 3–4-fold greater levels of AFB_1_ DNA adducts in mtDNA vs nDNA (94). In this model, the level of mtDNA damage was unchanged even at 24 h post exposure, with concomitant inhibition of transcription and translation (94,95). However, additional investigations using ICR mice or Syrian golden hamsters resulted in much lower levels of AFB_1_-induced mtDNA adducts, a result that could be attributed to inefficient transport of AFB_1_ into mitochondria, even though adequate levels of activating cytochrome P450s were present in hepatocytes (96). Overall, we conclude that the lack of an AFB_1_ mutational signature in mtDNA is likely a combination of lower overall adduct formation, combined with degradation of mtDNA containing AFB_1_ adducts due to blocked replication. These results are consistent with prior results reporting a lack of AFB_1_-induced mtDNA mutagenesis in *C. elegans* (97).

## Concluding remarks

This investigation demonstrated a modest aflatoxin-induced increase in the overall mutation frequency in NEIL1-deficient mice relative to repair-proficient mice, but without a change in spectra, while tumor frequencies and sizes were highly elevated. These data underscore the complexities of equating carcinogenesis as an endpoint with the magnitude of elevated mutagenesis. This conclusion is also supported by our data demonstrating that the equivalent levels of mutagenesis, as measured in males and females, do not correspond with the highly elevated tumor burden in male mice. Insights into key drivers of sex-specific cancers has recently been reported for colorectal cancers in which upregulation of Y-chromosome encoded histone demethylase KDM5D was shown to be largely responsible for the sex-specific differences in cancer rates and mortality (98). It is yet to be determined whether expression of this histone demethylase is a contributor to the sex-specific differences observed in HCC. Although the origins of the sex-specific differences in carcinogenesis may ultimately be different than sex-specific differences in the manifestation of metabolic syndrome in NEIL1-deficient mice, pathways regulating obesity, fatty liver disease, and insulin resistance may also have significant overlap with drivers of carcinogenesis.

## Supplementary Material

ugae006_Supplemental_File

## Data Availability

All sequencing data have been made available through the NCBI Sequence Read Archive (SRA) under BioProject accession number PRJNA1029184.
