# Supplementary material for: Frequencies and spectra of aflatoxin B1-induced mutations in liver genomes of NEIL1-deficient mice as revealed by duplex sequencing
Source: NAR Mol Med. 2024 May 17;1(2):ugae006. doi: 10.1093/narmme/ugae006 (PMC11105970; doi:10.1093/narmme/ugae006)
Supplement: ugae006_Supplemental_File [file ugae006_Supplemental_File.docx]

**Supplementary Information for**

**Frequencies and spectra of aflatoxin B_1_-induced mutations in liver genomes of NEIL1-deficient mice as revealed by duplex sequencing**

Irina G. Minko^1^†, Michael M. Luzadder^1^†, Vladimir L. Vartanian^1^, Sean P. M. Rice^1,2^, Megan M. Nguyen^3^, Monica Sanchez-Contreras^3^, Phu Van^4^, Scott R. Kennedy^3^, Amanda K. McCullough^1,5^, R. Stephen Lloyd^1,5^*

^1^Oregon Institute of Occupational Health Sciences, Oregon Health & Science University, Portland, Oregon, USA

^2^School of Public Health, Oregon Health & Science University - Portland State University, Portland, Oregon, USA

^3^Department of Laboratory Medicine and Pathology, University of Washington, Seattle, Washington, USA

^4^TwinStrand Biosciences, Inc., Seattle, Washington, USA

^5^Department of Molecular and Medical Genetics, Oregon Health & Science University, Portland, Oregon, USA

*To whom correspondence should be addressed. Tel: 1-503-494-9957; Email: [lloydst@ohsu.edu](mailto:lloydst@ohsu.edu)

†Contributed equally

**Content**

| ITEM | PAGE |
| --- | --- |
| **Assay performance metrics definitions.** | 3 |
| **Supplementary Table 1**. Assay Performance Metrics. Background mutations in nDNA. | 4 |
| **Supplementary Table 2**. Assay Performance Metrics. AFB_1_-induced mutations in nDNA. | 4 |
| **Supplementary Table 3**. Assay Performance Metrics. Spontaneous and AFB_1_-induced mutations in mtDNA. | 5 |
| **Supplementary Table 4.** Location of targets in mouse genome. | 6 |
| **Supplementary Table 5**. Types of mutations in individual samples in nDNA of unexposed mice: numbers and proportions. | 7 |
| **Supplementary Table 6**. Types of mutations in individual samples in nDNA of AFB_1_-exposed mice: numbers and proportions. | 8 |
| **Supplementary Table 7**. Types of mutations in individual samples in mtDNA of unexposed mice: numbers and proportions. | 10 |
| **Supplementary Table 8**. Types of mutations in individual samples in mtDNA of AFB_1_-exposed mice: numbers and proportions. | 11 |
| **Supplementary Table 9**. Frequencies of total mutations in nDNA. | 13 |
| **Supplementary Table 10**. Frequencies of SBS in nDNA. | 13 |
| **Supplementary Table 11**. Types of SBS in unexposed 6-mo mice in nDNA. | 14 |
| **Supplementary Table 12.** Types of SBS in AFB_1_-exposed 2.5-mo mice in nDNA. | 14 |
| **Supplementary Table 13**. Frequencies of total mutations in mtDNA. | 15 |
| **Supplementary Table 14**. Frequencies of SBS in mtDNA. | 15 |
| **Supplementary Table 15**. Types of SBS in unexposed 6-mo mice in mtDNA. | 16 |
| **Supplementary Table 16.** Types of SBS in AFB_1_-exposed 2.5-mo mice in mtDNA. | 16 |
| **Supplementary Table 17.** The trinucleotide mutation spectra (spontaneous mutations). | 17 |
| **Supplementary Table 18.** The trinucleotide mutation spectra (AFB_1_-induced mutations). | 20 |
| **Supplementary Table 19.** Distribution of mutations across genomic targets (spontaneous mutations). | 23 |
| **Supplementary Table 20.** Distribution of mutations across genomic targets (AFB_1_-induced mutations). | 24 |
| **Supplementary Table 21**. Mutation frequencies in nDNA targets grouped by location in either genic or intergenic region or by location. | 25 |
| **Supplementary Table 22.** Tumors observed in AFB_1_-exposed WT and *Neil1^-/-^* mice. | 26 |
| **Supplementary Figure 1**. Average DuplexSeq read depth per target. | 27 |
| **Supplementary Figure 2**. Frequencies and spectra of mutations in nDNA of WT and *Neil1^-/-^* mice: female versus male. | 28 |

**Supplementary Tables**

**Assay performance metrics definitions.**

Passing Filter Raw Reads: The total number of passing-filter sequencing reads.

Percent Selected Bases from Raw Reads: The fraction of passing-filter aligned bases that are located on or near the baited region.

Insert Size (Median with Standard Deviation): The median length (with standard deviation) of all inserts that are sequenced based on the alignment of the paired-end reads.

Mean and Maximum On-Target Duplex Depth: The mean and maximum coverage of the target region in the Duplex Consensus alignment file.

Peak Tag Family Size On-Target: The most frequent raw read family size across all single strand consensus that is greater than one. If there are multiple "most frequent" family sizes, then the smallest family is reported.

Informative Duplex Bases (non-N): The number of Duplex Consensus bases examined which is exclusive of any No-calls (ambiguous bases).

Percent Ns: The percent of No-calls (ambiguous bases) as determined from the total pool of filtered Duplex Consensus bases.

Average G.E. To Duplex Conversion: The percentage genome equivalents that are successfully converted into Duplex Consensus.

**Supplementary Table 1**. Assay Performance Metrics. Background mutations in nDNA.


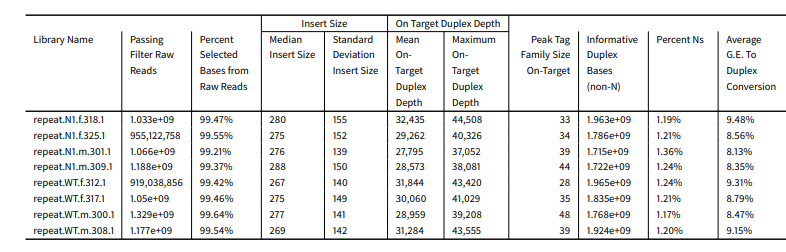


**Supplementary Table 2**. Assay Performance Metrics. AFB_1_-induced mutations in nDNA.


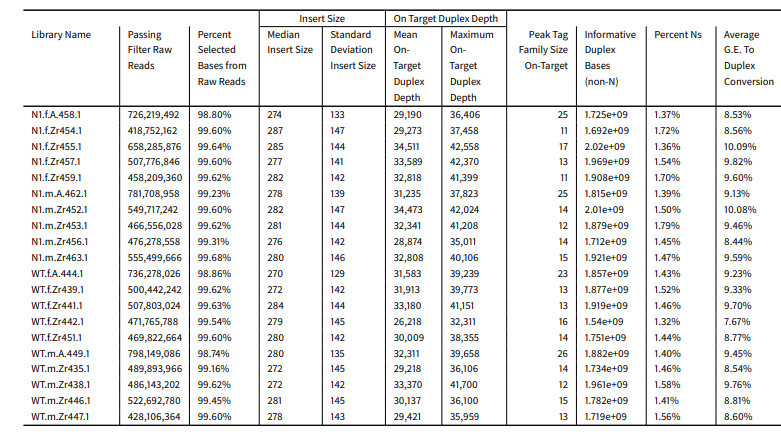


**Supplementary Table 3**. Assay Performance Metrics. Spontaneous and AFB_1_-induced mutations in mtDNA.


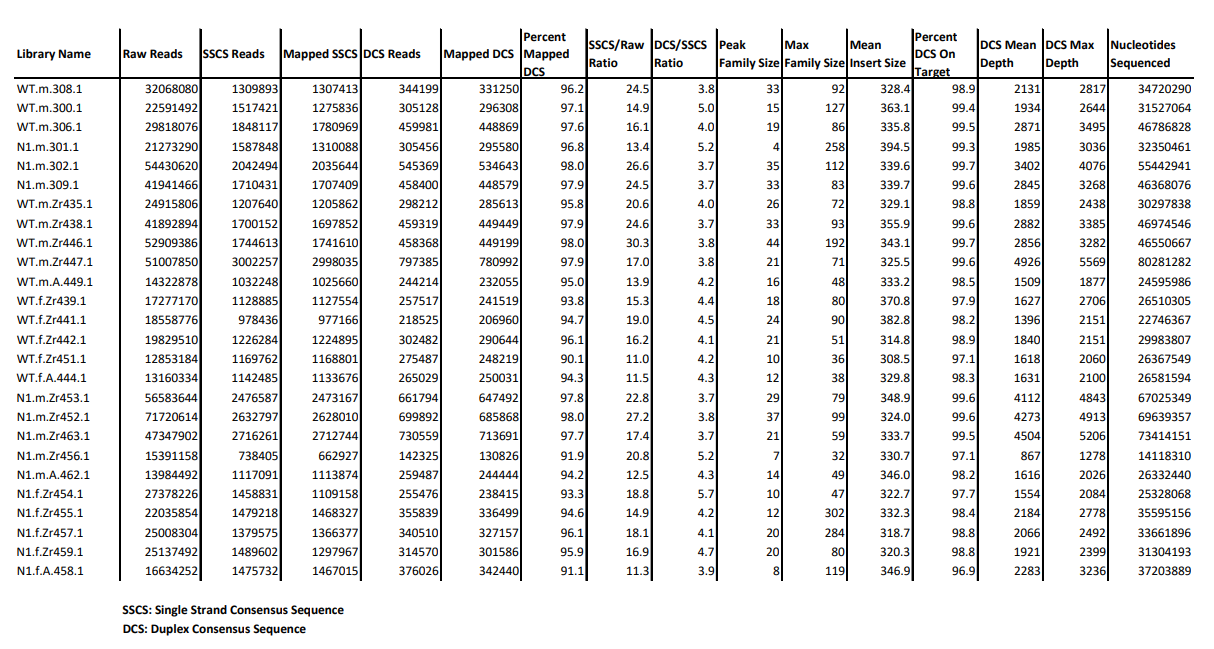


**Supplementary Table 4.** Location of targets in mouse genome.

| Target | Coordinates (genome reference mm10) | Genic Context |
| --- | --- | --- |
| chr1-1 | 69,304,217-69,306,617 | Intergenic |
| crh1-2 | 155,235,938-155,238,338 | Genic: intron in *BC034090* (orthologous to human *KIAA1614*) |
| chr2 | 50,833,175-50,835,575 | Intergenic |
| chr3 | 109,633,160-109,635,560 | Genic: intron in *Vav3* (Vav guanine nucleotide exchange factor 3) |
| chr4 | 96,825,280-96,827,680 | Intergenic |
| chr5 | 18,210,612-18,213,012 | Intergenic |
| chr6 | 119,170,706-119,173,106 | Genic: upstream *Dcp1b* (decapping mRNA 1B) |
| chr7 | 142,683,053-142,685,453 | Genic: upstream *Ins2* (insulin II) |
| chr8 | 43,954,521-43,956,921 | Intergenic |
| chr9 | 28,648,072-28,650,472 | Genic: intron in *Opcml* (opioid binding protein/cell adhesion molecule-like) |
| chr10 | 21,442,014-21,444,414 | Intergenic |
| chr11 | 37,934,364-37,936,764 | Intergenic |
| chr12 | 80,601,542-80,603,942 | Genic: intron, exon, and 3’ UTR *Galnt16* (polypeptide N-acetylgalactosaminyltransferase 16) |
| chr13 | 74,030,071-74,032,471 | Genic: intron, exon, and 3’ UTR  *Tppp* (tubulin polymerization promoting protein) |
| chr14 | 13,076,171-13,078,571 | Intergenic |
| chr15 | 66,779,762-66,782,162 | Genic: downstream *Sla* (src-like adaptor) |
| chr16 | 72,381,580-72,383,980 | Intergenic |
| chr17 | 94,009,028-94,011,428 | Intergenic |
| chr18 | 81,262,078-81,264,478 | Intergenic |
| chr19 | 4,618,813-4,621,213 | Genic: upstream *Lrfn4* (leucine rich repeat and fibronectin type III domain containing 4) and exon *Pcx* (pyruvate carboxylase) |

**Supplementary Table 5**. Types of mutations in individual samples in nDNA of unexposed mice: numbers and proportions (given in parentheses).

|  | C>A | C>G | C>T | T>A | T>C | T>G | indel | mnv | sv |
| --- | --- | --- | --- | --- | --- | --- | --- | --- | --- |
| WT females | | | | | | | | | |
| 312 | 11  (0.124) | 3  (0.034) | 33  (0.371) | 10  (0.112) | 13  (0.146) | 1  (0.011) | 15  (0.168) | 3  (0.034) | 0  (0.000) |
| 317 | 17  (0.173) | 8  (0.082) | 38  (0.388) | 9  (0.092) | 10  (0.102) | 3  (0.031) | 9  (0.092) | 3  (0.031) | 1  (0.010) |
| WT males | | | | | | | | | |
| 300 | 8  (0.123) | 3  (0.046) | 29  (0.446) | 7  (0.108) | 6  (0.092) | 5  (0.077) | 3  (0.046) | 4  (0.061) | 0  (0.000) |
| 308 | 13  (0.131) | 5  (0.050) | 28  (0.283) | 12  (0.121) | 12  (0.121) | 1  (0.010) | 22  (0.222) | 3  (0.030) | 3  (0.030) |
| *Neil1^-/-^* female | | | | | | | | | |
| 318 | 18  (0.617) | 2  (0.018) | 38  (0.352) | 13  (0.120) | 12  (0.111) | 5  (0.046) | 19  (0.176) | 1  (0.009) | 0  (0.000) |
| 325 | 17  (0.152) | 5  (0.044) | 41  (0.366) | 8  (0.071) | 15  (0.134) | 5  (0.045) | 15  (0.134) | 5  (0.045) | 1  (0.009) |
| *Neil1^-/-^* male | | | | | | | | | |
| 301 | 14  (0.141) | 4  (0.040) | 32  (0.323) | 8  (0.081) | 11  (0.111) | 6  (0.061) | 21  (0.212) | 3  (0.030) | 0  (0.000) |
| 309 | 14  (0.144) | 4  (0.041) | 31  (0.320) | 6  (0.062) | 10  (0.103) | 5  (0.051) | 21  (0.216) | 4  (0.041) | 2  (0.021) |

**Supplementary Table 6**. Types of mutations in individual samples in nDNA of AFB_1_-exposed mice: numbers and proportions (given in parentheses).

|  | C>A | C>G | C>T | T>A | T>C | T>G | indels | mnv | sv |
| --- | --- | --- | --- | --- | --- | --- | --- | --- | --- |
| WT females | | | | | | | | | |
| 439 | 1352  (0.688) | 191  (0.097) | 257  (0.131) | 46  (0.023) | 17  (0.009) | 10  (0.005) | 51  (0.026) | 38  (0.019) | 4  (0.002) |
| 441 | 1342  (0.676) | 176  (0.089) | 293  (0.148) | 57  (0.029) | 15  (0.008) | 8  (0.004) | 48  (0.024) | 38  (0.019) | 9  (0.005) |
| 442 | 1399  (0.701) | 172  (0.086) | 258  (0.129) | 64  (0.032) | 9  (0.005) | 11  (0.006) | 46  (0.023) | 28  (0.014) | 9  (0.005) |
| 444 | 1287  (0.665) | 173  (0.089) | 287  (0.148) | 47  (0.024) | 20  (0.010) | 12  (0.006) | 72  (0.037) | 29  (0.015) | 7  (0.004) |
| 451 | 1151  (0.674) | 162  (0.095) | 233  (0.136) | 50  (0.029) | 16  (0.009) | 15  (0.009) | 49  (0.029) | 21  (0.012) | 10  (0.006) |
| WT males | | | | | | | | | |
| 435 | 1313  (0.631) | 172  (0.083) | 302  (0.145) | 57  (0.027) | 11  (0.005) | 12  (0.006) | 185  (0.089) | 24  (0.012) | 4  (0.002) |
| 438 | 1466  (0.696) | 161  (0.076) | 294  (0.140) | 65  (0.031) | 27  (0.013) | 9  (0.004) | 48  (0.023) | 28  (0.013) | 9  (0.004) |
| 446 | 1388  (0.664) | 182  (0.087) | 291  (0.139) | 64  (0.031) | 22  (0.011) | 8  (0.004) | 106  (0.051) | 26  (0.012) | 4  (0.002) |
| 447 | 960  (0.695) | 109  (0.079) | 194  (0.140) | 40  (0.029) | 14  (0.010) | 8  (0.006) | 32  (0.023) | 17  (0.012) | 7  (0.005) |
| 449 | 1215  (0.651) | 158  (0.085) | 274  (0.147) | 75  (0.040) | 21  (0.011) | 8  (0.004) | 59  (0.032) | 38  (0.020) | 17  (0.009) |
| *Neil1^-/-^* female | | | | | | | | | |
| 454 | 1184  (0.664) | 131  (0.073) | 241  (0.135) | 100  (0.056) | 17  (0.010) | 18  (0.010) | 56  (0.031) | 33  (0.019) | 3  (0.002) |
| 455 | 1777  (0.665) | 241  (0.090) | 372  (0.139) | 144  (0.054) | 26  (0.010) | 17  (0.006) | 51  (0.019) | 41  (0.015) | 5  (0.002) |
| 457 | 1985  (0.684) | 242  (0.083) | 404  (0.139) | 142  (0.049) | 19  (0.007) | 16  (0.006) | 40  (0.014) | 45  (0.016) | 10  (0.003) |
| 458 | 1702  (0.675) | 205  (0.081) | 321  (0.127) | 116  (0.046) | 26  (0.010) | 27  (0.011) | 81  (0.032) | 34  (0.013) | 11  (0.004) |
| 459 | 1626  (0.673) | 216  (0.089) | 332  (0.137) | 111  (0.046) | 18  (0.007) | 22  (0.009) | 60  (0.025) | 26  (0.011) | 4  (0.002) |
| *Neil1^-/-^* male | | | | | | | | | |
| 452 | 1627  (0.664) | 210  (0.086) | 337  (0.138) | 122  (0.050) | 21  (0.009) | 21  (0.009) | 62  (0.025) | 43  (0.018) | 7  (0.003) |
| 453 | 1562  (0.682) | 192  (0.084) | 303  (0.132) | 103  (0.045) | 11  (0.005) | 16  (0.007) | 67  (0.029) | 23  (0.010) | 14  0.006 |
| 456 | 1555  (0.645) | 200  (0.083) | 315  (0.131) | 108  (0.045) | 16  (0.007) | 19  (0.008) | 154  (0.064) | 36  (0.015) | 6  (0.002) |
| 462 | 1278  (0.664) | 162  (0.084) | 290  (0.151) | 90  (0.047) | 11  (0.006) | 18  (0.009) | 48  (0.025) | 21  (0.011) | 8  (0.004) |
| 463 | 1460  (0.661) | 195  (0.088) | 294  (0.133) | 118  (0.053) | 25  (0.011) | 18  (0.008) | 67  (0.030) | 25  (0.011) | 7  (0.003) |

**Supplementary Table 7**. Types of mutations in individual samples in mtDNA of unexposed mice: numbers and proportions (given in parentheses).

|  | C > A | C > G | C > T | T > A | T > C | T > G | indels |
| --- | --- | --- | --- | --- | --- | --- | --- |
| WT males | | | | | | | |
| 300 | 24  (0.324) | 7  (0.095) | 29  (0.392) | 0  (0.000) | 8  (0.108) | 0  (0.000) | 6  (0.081) |
| 306 | 17  (0.198) | 4  (0.047) | 42  (0.488) | 0  (0.000) | 8  (0.093) | 0  (0.000) | 15  (0.174) |
| 308 | 17  (0.224) | 6  (0.079) | 30  (0.395) | 1  (0.013) | 9  (0.118) | 0  (0.000) | 13  (0.171) |
| *Neil1^-/-^* males | | | | | | | |
| 301 | 4  (0.082) | 3  (0.061) | 19  (0.388) | 0  (0.000) | 9  (0.184) | 0  (0.000) | 14  (0.286) |
| 302 | 23  (0.232) | 9  (0.091) | 39  (0.394) | 1  (0.010) | 13  (0.131) | 1  (0.010) | 13  (0.131) |
| 309 | 6  (0.067) | 1  (0.011) | 45  (0.506) | 2  (0.022) | 20  (0.225) | 1  (0.011) | 14  (0.157) |

**Supplementary Table 8**. Types of mutations in individual samples in mtDNA of AFB_1_-exposed mice: numbers and proportions (given in parentheses).

|  | | C>A | | C>G | C>T | T>A | T>C | T>G | indels |
| --- | --- | --- | --- | --- | --- | --- | --- | --- | --- |
| WT females | | | | | | | | | |
| 439 | | 2  (0.067) | | 3  (0.100) | 11  (0.367) | 1  (0.033) | 8  (0.267) | 1  (0.033) | 4  (0.133) |
| 441 | | 2  (0.118) | | 1  (0.059) | 3  (0.176) | 0  (0.000) | 1  (0.059) | 0  (0.000) | 10  (0.588) |
| 442 | | 14  (0.318) | | 4  (0.091) | 9  (0.205) | 0  (0.000) | 5  (0.114) | 3  (0.068) | 9  (0.205) |
| 444 | | 2  (0.065) | | 1  (0.032) | 17  (0.548) | 1  (0.032) | 5  (0.161) | 0  (0.000) | 5  (0.161) |
| 451 | | 6  (0.182) | | 2  (0.061) | 18  (0.545) | 0  (0.000) | 2  (0.061) | 0  (0.000) | 5  (0.152) |
| WT males | | | | | | | | | |
| 435 | | 17  (0.304) | | 3  (0.054) | 18  (0.321) | 0  (0.000) | 8  (0.143) | 0  (0.000) | 10  (0.179) |
| 438 | | 22  (0.328) | | 6  (0.090) | 20  (0.299) | 0  (0.000) | 10  (0.149) | 0  (0.000) | 9  (0.134) |
| 446 | | 19  (0.297) | | 4  0.063 | 23  0.359 | 3  0.047 | 3  0.047 | 0  0.000 | 12  0.188 |
| 447 | | 11  0.133 | | 12  0.145 | 35  0.422 | 3  0.036 | 9  0.108 | 0  0.000 | 13  0.157 |
| 449 | | 4  0.200 | | 0  0.000 | 7  0.350 | 0  0.000 | 3  0.150 | 0  0.000 | 6  0.300 |
| *Neil1^-/-^* female | | | | | | | | | |
| 454 | | 5  0.250 | | 1  0.050 | 6  0.300 | 0  0.000 | 5  0.250 | 0  0.000 | 3  0.150 |
| 455 | | 7  0.171 | | 2  0.049 | 14  0.341 | 1  0.024 | 10  0.244 | 0  0.000 | 7  0.171 |
| 457 | | 6  0.162 | | 4  0.108 | 12  0.324 | 2  0.054 | 5  0.135 | 0  0.000 | 8  0.216 |
| 458 | | 6  0.167 | | 4  0.111 | 16  0.444 | 0  0.000 | 5  0.139 | 0  0.000 | 5  0.139 |
| 459 | | 8  0.242 | | 3  0.091 | 13  0.394 | 0  0.000 | 2  0.061 | 0  0.000 | 7  0.212 |
| *Neil1^-/-^* male | | | | | | | | | |
| 452 | 11  0.155 | | 5  0.070 | | 26  0.366 | 2  0.028 | 13  0.183 | 1  0.014 | 13  0.183 |
| 453 | 8  0.125 | | 10  0.156 | | 27  0.422 | 0  0.000 | 7  0.109 | 0  0.000 | 12  0.188 |
| 456 | 4  0.174 | | 1  0.043 | | 7  0.304 | 1  0.043 | 5  0.217 | 0  0.000 | 5  0.217 |
| 462 | 0  0.000 | | 1  0.053 | | 9  0.474 | 1  0.053 | 0  0.000 | 1  0.053 | 7  0.368 |
| 463 | 4  0.056 | | 5  0.069 | | 33  0.458 | 3  0.042 | 9  0.125 | 0  0.000 | 18  0.250 |

**Supplementary Table 9**. Frequencies of total mutations in nDNA.

| Group of mice | Genotype | Mean MF | SE | Variance | Skewness |
| --- | --- | --- | --- | --- | --- |
| Unexposed 6-mo | WT | 5.97 × 10^-8^ | 0.48 × 10^-8^ | 9.2 × 10^-17^ | (-) 0.60 |
|  | *Neil1^-/-^* | 7.35 × 10^-8^ | 0.23 × 10^-8^ | 2.1 × 10^-17^ | 0.58 |
| AFB_1_-exposed 2.5-mo | WT | 1.30 × 10^-6^ | 0.05 × 10^-6^ | 3.0 × 10^-14^ | (-) 0.08 |
|  | *Neil1^-/-^* | 1.54 × 10^-6^ | 0.06 × 10^-6^ | 3.9 × 10^-14^ | 0.07 |

**Supplementary Table 10**. Frequencies of SBS in nDNA.

| Group of mice | Genotype | Mean MF | SE | Variance | Skewness |
| --- | --- | --- | --- | --- | --- |
| Unexposed 6-mo | WT | 4.86 × 10^-8^ | 0.37 × 10^-8^ | 5.3 × 10^-17^ | 0.75 |
|  | *Neil1^-/-^* | 5.71 × 10^-8^ | 0.28 × 10^-8^ | 3.1 × 10^-17^ | 0.48 |
| AFB_1_-exposed 2.5-mo | WT | 1.23 × 10^-6^ | 0.05 × 10^-6^ | 2.4 × 10^-14^ | 0.05 |
|  | *Neil1^-/-^* | 1.47 × 10^-6^ | 0.06 × 10^-6^ | 3.4 × 10^-14^ | 0.07 |

**Supplementary Table 11**. Types of SBS in unexposed 6-mo mice in nDNA.

| SBS type | Genotype | Mean MF | SE | Variance | Skewness |
| --- | --- | --- | --- | --- | --- |
| C > A | WT | 8.36 × 10^-9^ | 1.29 × 10^-9^ | 6.7 × 10^-18^ | 0.49 |
|  | *Neil1^-/-^* | 1.11 × 10^-8^ | 0.04 × 10^-8^ | 8.1 × 10^-19^ | 0.10 |
| C > G | WT | 3.25 × 10^-9^ | 0.82 × 10^-9^ | 2.7 × 10^-18^ | 0.75 |
|  | *Neil1^-/-^* | 2.69 × 10^-9^ | 0.49 × 10^-9^ | 9.6 × 10^-19^ | -0.85 |
| C > T | WT | 2.19 × 10^-8^ | 0.16 × 10^-8^ | 1.0 × 10^-17^ | 0.63 |
|  | *Neil1^-/-^* | 2.51 × 10^-8^ | 0.14 × 10^-8^ | 8.3 × 10^-18^ | 0.90 |
| T > A | WT | 6.45 × 10^-9^ | 0.61 × 10^-9^ | 1.5 × 10^-18^ | 0.18 |
|  | *Neil1^-/-^* | 6.11 × 10^-9^ | 0.83 × 10^-9^ | 2.7 × 10^-18^ | 0.52 |
| T > C | WT | 6.94 × 10^-9^ | 0.93 × 10^-9^ | 3.5 × 10^-18^ | -0.77 |
|  | *Neil1^-/-^* | 8.48 × 10^-9^ | 0.76 × 10^-9^ | 2.3 × 10^-18^ | 0.93 |
| T > G | WT | 1.75 × 10^-9^ | 0.70 × 10^-9^ | 2.0 × 10^-18^ | 0.53 |
|  | *Neil1^-/-^* | 3.73 × 10^-9^ | 0.27 × 10^-9^ | 3.0 × 10^-19^ | 0.74 |

**Supplementary Table 12.** Types of SBS in AFB_1_-exposed 2.5-mo mice in nDNA.

| SBS type | Genotype | Mean MF | SE | Variance | Skewness |
| --- | --- | --- | --- | --- | --- |
| C > A | WT | 8.76 × 10^-7^ | 0.37 × 10^-7^ | 1.4 × 10^-14^ | 0.37 |
|  | *Neil1^-/-^* | 1.03 × 10^-6^ | 0.04 × 10^-6^ | 1.8 × 10^-14^ | 0.13 |
| C > G | WT | 1.13 × 10^-7^ | 0.05 × 10^-7^ | 2.8 × 10^-16^ | -0.72 |
|  | *Neil1^-/-^* | 1.30 × 10^-7^ | 0.06 × 10^-7^ | 3.4 × 10^-16^ | -0.74 |
| C > T | WT | 1.82 × 10^-7^ | 0.07 × 10^-7^ | 5.3 × 10^-16^ | -0.45 |
|  | *Neil1^-/-^* | 2.09 × 10^-7^ | 0.08 × 10^-7^ | 5.7 × 10^-16^ | 0.13 |
| T > A | WT | 3.85 × 10^-8^ | 0.25 × 10^-8^ | 6.1 × 10^-17^ | 0.24 |
|  | *Neil1^-/-^* | 7.52 × 10^-8^ | 0.28 × 10^-8^ | 8.0 × 10^-17^ | -0.04 |
| T > C | WT | 1.15 × 10^-8^ | 0.10 × 10^-8^ | 1.0 × 10^-17^ | 0.24 |
|  | *Neil1^-/-^* | 1.24 × 10^-8^ | 0.11 × 10^-8^ | 1.3 × 10^-17^ | 0.07 |
| T > G | WT | 6.92 × 10^-9^ | 0.59 × 10^-9^ | 3.5 × 10^-18^ | 0.64 |
|  | *Neil1^-/-^* | 1.26 × 10^-8^ | 0.09 × 10^-8^ | 7.5 × 10^-18^ | 1.4 |

**Supplementary Table 13**. Frequencies of total mutations in mtDNA.

| Group of mice | Genotype | Mean MF | SE | Variance | Skewness |
| --- | --- | --- | --- | --- | --- |
| Unexposed 6-mo | WT | 2.12 × 10^-6^ | 1.50 × 10^-7^ | 6.8 × 10^-14^ | -0.43 |
|  | *Neil1^-/-^* | 1.74 × 10^-6^ | 1.19 × 10^-7^ | 4.3 × 10^-14^ | -0.39 |
| AFB_1_-exposed 2.5-mo | WT | 1.23 × 10^-6^ | 1.03 × 10^-7^ | 1.1 × 10^-13^ | 0.26 |
|  | *Neil1^-/-^* | 1.04 × 10^-6^ | 7.77 × 10^-8^ | 6.0 × 10^-14^ | 1.28 |

**Supplementary Table 14**. Frequencies of SBS in mtDNA.

| Group of mice | Genotype | Mean MF | SE | Variance | Skewness |
| --- | --- | --- | --- | --- | --- |
| Unexposed 6-mo | WT | 1.83 × 10^-6^ | 1.85 × 10^-7^ | 1.0 × 10^-13^ | 0.09 |
|  | *Neil1^-/-^* | 1.42 × 10^-6^ | 1.69 × 10^-7^ | 8.5 × 10^-14^ | 0.67 |
| AFB_1_-exposed 2.5-mo | WT | 9.81 × 10^-7^ | 1.08 × 10^-7^ | 1.2 × 10^-13^ | 0.57 |
|  | *Neil1^-/-^* | 8.23 × 10^-7^ | 6.59 × 10^-8^ | 4.4 × 10^-14^ | 0.53 |

**Supplementary Table 15**. Types of SBS in unexposed 6-mo mice in mtDNA.

| SBS type | Genotype | Mean MF | SE | Variance | Skewness |
| --- | --- | --- | --- | --- | --- |
| C > A | WT | 5.38 × 10^-7^ | 1.17 × 10^-7^ | 4.1 × 10^-14^ | 0.42 |
|  | *Neil1^-/-^* | 2.23 × 10^-7^ | 9.62 × 10^-8^ | 2.8 × 10^-14^ | 0.71 |
| C > G | WT | 1.60 × 10^-7^ | 3.99 × 10^-8^ | 4.8 × 10^-15^ | -0.33 |
|  | *Neil1^-/-^* | 9.22 × 10^-8^ | 4.06 × 10^-8^ | 5.0 × 10^-15^ | -0.01 |
| C > T | WT | 8.94 × 10^-7^ | 1.62 × 10^-8^ | 7.9 × 10^-16^ | -0.25 |
|  | *Neil1^-/-^* | 7.54 × 10^-7^ | 1.13 × 10^-7^ | 3.9 × 10^-14^ | 0.44 |
| T > A | WT | 9.60 × 10^-9^ | 9.60 × 10^-9^ | 2.8 × 10^-16^ | 0.71 |
|  | *Neil1^-/-^* | 2.04 × 10^-8^ | 1.25 × 10^-8^ | 4.7 × 10^-16^ | 0.20 |
| T > C | WT | 2.28 × 10^-7^ | 2.85 × 10^-8^ | 2.4 × 10^-15^ | -0.70 |
|  | *Neil1^-/-^* | 3.15 × 10^-7^ | 5.97 × 10^-8^ | 1.1 × 10^-14^ | 0.57 |
| T > G | WT | 0 | 0 | 0 | NA |
|  | *Neil1^-/-^* | 1.32 × 10^-8^ | 6.68 × 10^-9^ | 1.3 × 10^-16^ | -0.63 |
| indels | WT | 2.95 × 10^-7^ | 5.47 × 10^-8^ | 9.0 × 10^-15^ | -0.46 |
|  | *Neil1^-/-^* | 3.23 × 10^-7^ | 5.82 × 10^-8^ | 1.0 × 10^-14^ | 0.37 |

**Supplementary Table 16.** Types of SBS in AFB_1_-exposed 2.5-mo mice in mtDNA.

| SBS type | Genotype | Mean MF | SE | Variance | Skewness |
| --- | --- | --- | --- | --- | --- |
| C > A | WT | 2.67 × 10^-7^ | 5.98 × 10^-8^ | 3.6 × 10^-14^ | 0.37 |
|  | *Neil1^-/-^* | 1.60 × 10^-7^ | 2.70 × 10^-8^ | 7.3 × 10^-15^ | -0.48 |
| C > G | WT | 8.66 × 10^-8^ | 1.51 × 10^-8^ | 2.3 × 10^-15^ | -0.44 |
|  | *Neil1^-/-^* | 8.16 × 10^-8^ | 1.13 × 10^-8^ | 1.3 × 10^-15^ | 0.52 |
| C > T | WT | 4.40 × 10^-7^ | 5.42 × 10^-8^ | 2.9 × 10^-14^ | -0.23 |
|  | *Neil1^-/-^* | 3.90 × 10^-7^ | 2.22 × 10^-8^ | 4.9 × 10^-15^ | -0.75 |
| T > A | WT | 1.77 × 10^-8^ | 7.64 × 10^-9^ | 5.8 × 10^-16^ | 0.77 |
|  | *Neil1^-/-^* | 2.66 × 10^-8^ | 8.30 × 10^-9^ | 6.9 × 10^-16^ | 0.33 |
| T > C | WT | 1.55 × 10^-7^ | 2.74 × 10^-8^ | 7.5 × 10^-15^ | 0.35 |
|  | *Neil1^-/-^* | 1.59 × 10^-7^ | 3.24 × 10^-8^ | 1.1 × 10^-14^ | 0.45 |
| T > G | WT | 1.38× 10^-8^ | 1.03 × 10^-8^ | 1.1 × 10^-15^ | 2.2 |
|  | *Neil1^-/-^* | 5.23 × 10^-9^ | 3.91 × 10^-9^ | 1.5 × 10^-16^ | 2.2 |
| indels | WT | 2.45 × 10^-7^ | 2.85 × 10^-8^ | 8.1 × 10^-15^ | 0.98 |
|  | *Neil1^-/-^* | 2.14 × 10^-7^ | 2.15 × 10^-8^ | 4.6 × 10^-15^ | 0.55 |

**Supplementary Table 17.** The trinucleotide mutation spectra (spontaneous mutations).

| Trinucleotide context | Mutation subtype | WT | | Neil1 ^-/-^ | |
| --- | --- | --- | --- | --- | --- |
|  |  | Mean proportion | SE | Mean proportion | SE |
| ACA | C>A | 7.07 × 10^-3^ | 2.93 × 10^-3^ | 1.13 × 10^-2^ | 4.39 × 10^-3^ |
| ACC | C>A | 6.97 × 10^-3^ | 4.19 × 10^-3^ | 5.02 × 10^-3^ | 2.97 × 10^-3^ |
| ACG | C>A | 2.34 × 10^-2^ | 1.39 × 10^-2^ | 0.00 | 0.00 |
| ACT | C>A | 0.00 | 0.00 | 8.85 × 10^-3^ | 5.11 × 10^-3^ |
| CCA | C>A | 8.99 × 10^-3^ | 3.51 × 10^-3^ | 9.08 × 10^-3^ | 5.45 × 10^-3^ |
| CCC | C>A | 4.85 × 10^-3^ | 4.85 × 10^-3^ | 1.05 × 10^-2^ | 4.20 × 10^-3^ |
| CCG | C>A | 0.00 | 0.00 | 1.15 × 10^-2^ | 1.15 × 10^-2^ |
| CCT | C>A | 4.92 × 10^-3^ | 4.92 × 10^-3^ | 5.67 × 10^-3^ | 3.70 × 10^-3^ |
| GCA | C>A | 1.19 × 10^-2^ | 2.60 × 10^-3^ | 6.45 × 10^-3^ | 2.24 × 10^-3^ |
| GCC | C>A | 1.58 × 10^-2^ | 6.51 × 10^-3^ | 8.05 × 10^-3^ | 5.72 × 10^-3^ |
| GCG | C>A | 0.00 | 0.00 | 0.00 | 0.00 |
| GCT | C>A | 1.01 × 10^-2^ | 5.47 × 10^-3^ | 1.95 × 10^-2^ | 5.07 × 10^-3^ |
| TCA | C>A | 5.64 × 10^-3^ | 2.00 × 10^-3^ | 7.59 × 10^-3^ | 2.88 × 10^-3^ |
| TCC | C>A | 3.51 × 10^-3^ | 2.08 × 10^-3^ | 1.40 × 10^-2^ | 5.41 × 10^-3^ |
| TCG | C>A | 0.00 | 0.00 | 3.47 × 10^-2^ | 2.01 × 10^-2^ |
| TCT | C>A | 1.24 × 10^-2^ | 3.05 × 10^-3^ | 9.77 × 10^-3^ | 1.33 × 10^-3^ |
| ACA | C>G | 4.14 × 10^-3^ | 2.46 × 10^-3^ | 6.23 × 10^-3^ | 2.17 × 10^-3^ |
| ACC | C>G | 4.19 × 10^-3^ | 4.19 × 10^-3^ | 0.00 | 0.00 |
| ACG | C>G | 0.00 | 0.00 | 0.00 | 0.00 |
| ACT | C>G | 5.30 × 10^-3^ | 1.84 × 10^-3^ | 2.05 × 10^-3^ | 2.05 × 10^-3^ |
| CCA | C>G | 6.82 × 10^-3^ | 2.41 × 10^-3^ | 1.82 × 10^-3^ | 1.82 × 10^-3^ |
| CCC | C>G | 1.78 × 10^-3^ | 1.78 × 10^-3^ | 0.00 | 0.00 |
| CCG | C>G | 0.00 | 0.00 | 0.00 | 0.00 |
| CCT | C>G | 1.23 × 10^-3^ | 1.23 × 10^-3^ | 5.15 × 10^-3^ | 3.66 × 10^-3^ |
| GCA | C>G | 2.24 × 10^-3^ | 2.24 × 10^-3^ | 4.06 × 10^-3^ | 2.41 × 10^-3^ |
| GCC | C>G | 2.62 × 10^-3^ | 2.62 × 10^-3^ | 0.00 | 0.00 |
| GCG | C>G | 0.00 | 0.00 | 0.00 | 0.00 |
| GCT | C>G | 0.00 | 0.00 | 2.24 × 10^-3^ | 2.24 × 10^-3^ |
| TCA | C>G | 0.00 | 0.00 | 0.00 | 0.00 |
| TCC | C>G | 0.00 | 0.00 | 2.33 × 10^-3^ | 2.33 × 10^-3^ |
| TCG | C>G | 0.00 | 0.00 | 0.00 | 0.00 |
| TCT | C>G | 6.03 × 10^-3^ | 9.23 × 10^-4^ | 2.56 × 10^-3^ | 1.52 × 10^-3^ |
| ACA | C>T | 3.85 × 10^-2^ | 1.97 × 10^-2^ | 3.11 × 10^-2^ | 1.40 × 10^-3^ |
| ACC | C>T | 1.73 × 10^-2^ | 5.79 × 10^-3^ | 2.00 × 10^-2^ | 8.98 × 10^-3^ |
| ACG | C>T | 8.78 × 10^-2^ | 3.39 × 10^-2^ | 1.44 × 10^-01^ | 1.40 × 10^-2^ |
| ACT | C>T | 1.15 × 10^-2^ | 1.53 × 10^-3^ | 1.89 × 10^-2^ | 9.13 × 10^-3^ |
| CCA | C>T | 2.32 × 10^-2^ | 2.63 × 10^-3^ | 2.85 × 10^-2^ | 4.76 × 10^-3^ |
| CCC | C>T | 1.46 × 10^-2^ | 6.04 × 10^-3^ | 2.31 × 10^-2^ | 5.97 × 10^-3^ |
| CCG | C>T | 1.53 × 10^-01^ | 4.87 × 10^-2^ | 3.39 × 10^-2^ | 1.96 × 10^-2^ |
| CCT | C>T | 2.77 × 10^-2^ | 7.63 × 10^-3^ | 1.05 × 10^-2^ | 4.86 × 10^-3^ |
| GCA | C>T | 7.32 × 10^-3^ | 2.83 × 10^-3^ | 1.23 × 10^-2^ | 4.80 × 10^-3^ |
| GCC | C>T | 1.70 × 10^-2^ | 6.45 × 10^-3^ | 1.11 × 10^-2^ | 7.85 × 10^-3^ |
| GCG | C>T | 1.12 × 10^-01^ | 4.35 × 10^-2^ | 6.38 × 10^-2^ | 2.25 × 10^-2^ |
| GCT | C>T | 9.02 × 10^-3^ | 1.37 × 10^-3^ | 1.11 × 10^-2^ | 5.32 × 10^-3^ |
| TCA | C>T | 6.91 × 10^-3^ | 1.06 × 10^-3^ | 1.43 × 10^-2^ | 3.47 × 10^-4^ |
| TCC | C>T | 1.47 × 10^-2^ | 6.15 × 10^-3^ | 1.92 × 10^-2^ | 1.73 × 10^-3^ |
| TCG | C>T | 1.16 × 10^-01^ | 2.59 × 10^-2^ | 1.55 × 10^-01^ | 4.51 × 10^-2^ |
| TCT | C>T | 9.96 × 10^-3^ | 2.55 × 10^-3^ | 1.95 × 10^-2^ | 2.67 × 10^-3^ |
| ATA | T>A | 8.69 × 10^-3^ | 1.35 × 10^-3^ | 8.51 × 10^-3^ | 5.97 × 10^-3^ |
| ATC | T>A | 5.76 × 10^-3^ | 3.71 × 10^-3^ | 0.00 | 0.00 |
| ATG | T>A | 4.40 × 10^-3^ | 2.63 × 10^-3^ | 9.00 × 10^-3^ | 2.41 × 10^-3^ |
| ATT | T>A | 8.15 × 10^-3^ | 4.92 × 10^-3^ | 1.21 × 10^-3^ | 1.21 × 10^-3^ |
| CTA | T>A | 2.46 × 10^-3^ | 2.46 × 10^-3^ | 5.27 × 10^-3^ | 5.27 × 10^-3^ |
| CTC | T>A | 7.25 × 10^-3^ | 3.24 × 10^-3^ | 5.50 × 10^-3^ | 3.82 × 10^-3^ |
| CTG | T>A | 2.16 × 10^-3^ | 2.16 × 10^-3^ | 2.66 × 10^-3^ | 1.58 × 10^-3^ |
| CTT | T>A | 9.99 × 10^-3^ | 3.09 × 10^-3^ | 9.04 × 10^-3^ | 3.22 × 10^-3^ |
| GTA | T>A | 0.00 | 0.00 | 3.37 × 10^-3^ | 3.37 × 10^-3^ |
| GTC | T>A | 8.01 × 10^-3^ | 5.53 × 10^-3^ | 3.12 × 10^-3^ | 3.12 × 10^-3^ |
| GTG | T>A | 6.84 × 10^-3^ | 2.65 × 10^-3^ | 2.46 × 10^-3^ | 2.46 × 10^-3^ |
| GTT | T>A | 4.10 × 10^-3^ | 2.42 × 10^-3^ | 1.16 × 10^-2^ | 4.62 × 10^-3^ |
| TTA | T>A | 2.71 × 10^-3^ | 2.71 × 10^-3^ | 3.78 × 10^-3^ | 2.18 × 10^-3^ |
| TTC | T>A | 0.00 | 0.00 | 0.00 | 0.00 |
| TTG | T>A | 0.00 | 0.00 | 0.00 | 0.00 |
| TTT | T>A | 3.41 × 10^-3^ | 2.16 × 10^-3^ | 2.05 × 10^-3^ | 1.21 × 10^-3^ |
| ATA | T>C | 1.02 × 10^-2^ | 6.16 × 10^-3^ | 8.15 × 10^-3^ | 7.07 × 10^-4^ |
| ATC | T>C | 1.17 × 10^-2^ | 4.49 × 10^-3^ | 0.00 | 0.00 |
| ATG | T>C | 6.60 × 10^-3^ | 5.06 × 10^-3^ | 0.00 | 0.00 |
| ATT | T>C | 3.49 × 10^-3^ | 3.49 × 10^-3^ | 6.34 × 10^-3^ | 2.86 × 10^-3^ |
| CTA | T>C | 2.53 × 10^-3^ | 2.53 × 10^-3^ | 9.14 × 10^-3^ | 3.20 × 10^-3^ |
| CTC | T>C | 3.88 × 10^-3^ | 2.24 × 10^-3^ | 2.23 × 10^-3^ | 2.23 × 10^-3^ |
| CTG | T>C | 5.52 × 10^-3^ | 2.43 × 10^-3^ | 9.35 × 10^-3^ | 3.74 × 10^-3^ |
| CTT | T>C | 4.47 × 10^-3^ | 2.87 × 10^-3^ | 1.23 × 10^-2^ | 2.93 × 10^-3^ |
| GTA | T>C | 1.34 × 10^-2^ | 5.55 × 10^-3^ | 1.33 × 10^-2^ | 7.69 × 10^-3^ |
| GTC | T>C | 2.15 × 10^-3^ | 2.15 × 10^-3^ | 3.12 × 10^-3^ | 3.12 × 10^-3^ |
| GTG | T>C | 1.27 × 10^-2^ | 1.80 × 10^-3^ | 3.84 × 10^-3^ | 2.28 × 10^-3^ |
| GTT | T>C | 5.33 × 10^-3^ | 3.44 × 10^-3^ | 6.10 × 10^-3^ | 3.64 × 10^-3^ |
| TTA | T>C | 1.78 × 10^-3^ | 1.78 × 10^-3^ | 2.69 × 10^-3^ | 2.69 × 10^-3^ |
| TTC | T>C | 0.00 | 0.00 | 2.37 × 10^-3^ | 2.37 × 10^-3^ |
| TTG | T>C | 3.88 × 10^-3^ | 2.24 × 10^-3^ | 4.29 × 10^-3^ | 2.48 × 10^-3^ |
| TTT | T>C | 0.00 | 0.00 | 5.42 × 10^-3^ | 2.01 × 10^-3^ |
| ATA | T>G | 0.00 | 0.00 | 4.44 × 10^-3^ | 2.57 × 10^-3^ |
| ATC | T>G | 3.89 × 10^-3^ | 3.89 × 10^-3^ | 2.70 × 10^-3^ | 2.70 × 10^-3^ |
| ATG | T>G | 2.62 × 10^-3^ | 2.62 × 10^-3^ | 3.86 × 10^-3^ | 2.23 × 10^-3^ |
| ATT | T>G | 1.16 × 10^-3^ | 1.16 × 10^-3^ | 7.12 × 10^-3^ | 3.04 × 10^-3^ |
| CTA | T>G | 0.00 | 0.00 | 0.00 | 0.00 |
| CTC | T>G | 0.00 | 0.00 | 0.00 | 0.00 |
| CTG | T>G | 0.00 | 0.00 | 1.56 × 10^-3^ | 1.56 × 10^-3^ |
| CTT | T>G | 6.59 × 10^-3^ | 4.24 × 10^-3^ | 8.60 × 10^-3^ | 1.38 × 10^-3^ |
| GTA | T>G | 0.00 | 0.00 | 0.00 | 0.00 |
| GTC | T>G | 0.00 | 0.00 | 4.43 × 10^-3^ | 4.43 × 10^-3^ |
| GTG | T>G | 0.00 | 0.00 | 0.00 | 0.00 |
| GTT | T>G | 5.97 × 10^-3^ | 3.59 × 10^-3^ | 2.54 × 10^-3^ | 2.54 × 10^-3^ |
| TTA | T>G | 0.00 | 0.00 | 1.91 × 10^-3^ | 1.91 × 10^-3^ |
| TTC | T>G | 0.00 | 0.00 | 0.00 | 0.00 |
| TTG | T>G | 0.00 | 0.00 | 1.46 × 10^-3^ | 1.46 × 10^-3^ |
| TTT | T>G | 1.16 × 10^-3^ | 1.16 × 10^-3^ | 0.00 | 0.00 |

**Supplementary Table 18.** The trinucleotide mutation spectra (AFB_1_-induced mutations).

| Trinucleotide context | Mutation subtype | WT | | Neil1 ^-/-^ | |
| --- | --- | --- | --- | --- | --- |
|  |  | Mean proportion | SE | Mean proportion | SE |
| ACA | C>A | 1.35 × 10^-2^ | 6.18 × 10^-4^ | 1.37 × 10^-2^ | 7.25 × 10^-4^ |
| ACC | C>A | 1.11 × 10^-2^ | 5.74 × 10^-4^ | 1.08 × 10^-2^ | 8.07 × 10^-4^ |
| ACG | C>A | 3.72 × 10^-2^ | 3.47 × 10^-3^ | 4.34 × 10^-2^ | 1.82 × 10^-3^ |
| ACT | C>A | 1.26 × 10^-2^ | 5.91 × 10^-4^ | 1.18 × 10^-2^ | 6.41 × 10^-4^ |
| CCA | C>A | 2.56 × 10^-2^ | 6.87 × 10^-4^ | 2.73 × 10^-2^ | 9.26 × 10^-4^ |
| CCC | C>A | 2.78 × 10^-2^ | 9.09 × 10^-4^ | 2.57 × 10^-2^ | 9.00 × 10^-4^ |
| CCG | C>A | 7.64 × 10^-2^ | 3.97 × 10^-3^ | 7.11 × 10^-2^ | 2.37 × 10^-3^ |
| CCT | C>A | 1.98 × 10^-2^ | 5.77 × 10^-4^ | 2.20 × 10^-2^ | 5.64 × 10^-4^ |
| GCA | C>A | 6.81 × 10^-2^ | 1.24 × 10^-3^ | 7.24 × 10^-2^ | 1.34 × 10^-3^ |
| GCC | C>A | 6.53 × 10^-2^ | 1.34 × 10^-3^ | 6.73 × 10^-2^ | 1.12 × 10^-3^ |
| GCG | C>A | 2.03 × 10^-1^ | 4.80 × 10^-3^ | 1.87 × 10^-1^ | 3.55 × 10^-3^ |
| GCT | C>A | 5.57 × 10^-2^ | 1.04 × 10^-3^ | 5.91 × 10^-2^ | 1.01 × 10^-3^ |
| TCA | C>A | 1.73 × 10^-2^ | 7.60 × 10^-4^ | 1.77 × 10^-2^ | 4.66 × 10^-4^ |
| TCC | C>A | 2.28 × 10^-2^ | 8.85 × 10^-4^ | 2.22 × 10^-2^ | 1.16 × 10^-3^ |
| TCG | C>A | 4.50 × 10^-2^ | 2.87 × 10^-3^ | 4.36 × 10^-2^ | 2.50 × 10^-3^ |
| TCT | C>A | 1.29 × 10^-2^ | 4.65 × 10^-4^ | 1.50 × 10^-2^ | 3.44 × 10^-4^ |
| ACA | C>G | 1.82 × 10^-3^ | 1.60 × 10^-4^ | 2.13 × 10^-3^ | 2.19 × 10^-4^ |
| ACC | C>G | 1.69 × 10^-3^ | 2.38 × 10^-4^ | 1.49 × 10^-3^ | 2.11 × 10^-4^ |
| ACG | C>G | 3.02 × 10^-3^ | 6.63 × 10^-4^ | 4.84 × 10^-3^ | 6.30 × 10^-4^ |
| ACT | C>G | 1.38 × 10^-3^ | 2.08 × 10^-4^ | 1.16 × 10^-3^ | 2.44 × 10^-4^ |
| CCA | C>G | 2.23 × 10^-3^ | 3.16 × 10^-4^ | 2.72 × 10^-3^ | 1.59 × 10^-4^ |
| CCC | C>G | 2.95 × 10^-3^ | 3.75 × 10^-4^ | 2.59 × 10^-3^ | 2.36 × 10^-4^ |
| CCG | C>G | 7.69 × 10^-3^ | 1.44 × 10^-3^ | 6.95 × 10^-3^ | 1.03 × 10^-3^ |
| CCT | C>G | 1.28 × 10^-3^ | 1.89 × 10^-4^ | 1.91 × 10^-3^ | 2.11 × 10^-4^ |
| GCA | C>G | 6.16 × 10^-3^ | 3.72 × 10^-4^ | 6.76 × 10^-3^ | 4.51 × 10^-4^ |
| GCC | C>G | 1.49 × 10^-2^ | 4.27 × 10^-4^ | 1.31 × 10^-2^ | 5.15 × 10^-4^ |
| GCG | C>G | 3.14 × 10^-2^ | 1.75 × 10^-3^ | 2.95 × 10^-2^ | 2.10 × 10^-3^ |
| GCT | C>G | 7.56 × 10^-3^ | 3.36 × 10^-4^ | 8.11 × 10^-3^ | 3.96 × 10^-4^ |
| TCA | C>G | 2.22 × 10^-3^ | 1.68 × 10^-4^ | 2.31 × 10^-3^ | 1.74 × 10^-4^ |
| TCC | C>G | 2.49 × 10^-3^ | 2.27 × 10^-4^ | 2.25 × 10^-3^ | 2.29 × 10^-4^ |
| TCG | C>G | 8.14 × 10^-3^ | 1.21 × 10^-3^ | 7.96 × 10^-3^ | 1.32 × 10^-3^ |
| TCT | C>G | 1.89 × 10^-3^ | 1.29 × 10^-4^ | 1.95 × 10^-3^ | 1.52 × 10^-4^ |
| ACA | C>T | 2.88 × 10^-3^ | 2.97 × 10^-4^ | 2.80 × 10^-3^ | 2.31 × 10^-4^ |
| ACC | C>T | 2.83 × 10^-3^ | 3.15 × 10^-4^ | 2.71 × 10^-3^ | 2.90 × 10^-4^ |
| ACG | C>T | 1.27 × 10^-2^ | 1.65 × 10^-3^ | 9.67 × 10^-3^ | 1.67 × 10^-3^ |
| ACT | C>T | 1.55 × 10^-3^ | 2.24 × 10^-4^ | 2.55 × 10^-3^ | 2.40 × 10^-4^ |
| CCA | C>T | 3.20 × 10^-3^ | 4.47 × 10^-4^ | 3.40 × 10^-3^ | 2.91 × 10^-4^ |
| CCC | C>T | 4.34 × 10^-3^ | 4.09 × 10^-4^ | 3.85 × 10^-3^ | 2.53 × 10^-4^ |
| CCG | C>T | 9.44 × 10^-3^ | 1.65 × 10^-3^ | 1.02 × 10^-2^ | 1.53 × 10^-3^ |
| CCT | C>T | 2.87 × 10^-3^ | 2.07 × 10^-4^ | 3.53 × 10^-3^ | 2.32 × 10^-4^ |
| GCA | C>T | 1.28 × 10^-2^ | 8.39 × 10^-4^ | 1.24 × 10^-2^ | 6.32 × 10^-4^ |
| GCC | C>T | 1.23 × 10^-2^ | 1.06 × 10^-3^ | 1.11 × 10^-2^ | 6.09 × 10^-4^ |
| GCG | C>T | 5.13 × 10^-2^ | 3.90 × 10^-3^ | 4.57 × 10^-2^ | 3.67 × 10^-3^ |
| GCT | C>T | 9.90 × 10^-3^ | 6.35 × 10^-4^ | 1.10 × 10^-2^ | 2.22 × 10^-4^ |
| TCA | C>T | 4.92 × 10^-3^ | 3.67 × 10^-4^ | 4.65 × 10^-3^ | 2.37 × 10^-4^ |
| TCC | C>T | 7.99 × 10^-3^ | 5.43 × 10^-4^ | 7.81 × 10^-3^ | 4.46 × 10^-4^ |
| TCG | C>T | 2.07 × 10^-2^ | 2.26 × 10^-3^ | 2.05 × 10^-2^ | 1.31 × 10^-3^ |
| TCT | C>T | 4.64 × 10^-3^ | 3.12 × 10^-4^ | 5.33 × 10^-3^ | 2.11 × 10^-4^ |
| ATA | T>A | 5.32 × 10^-4^ | 1.31 × 10^-4^ | 7.19 × 10^-4^ | 1.26 × 10^-4^ |
| ATC | T>A | 4.06 × 10^-4^ | 1.15 × 10^-4^ | 9.20 × 10^-4^ | 1.66 × 10^-4^ |
| ATG | T>A | 1.08 × 10^-3^ | 1.62 × 10^-4^ | 1.11 × 10^-3^ | 1.61 × 10^-4^ |
| ATT | T>A | 3.16 × 10^-4^ | 7.60 × 10^-5^ | 4.67 × 10^-4^ | 1.17 × 10^-4^ |
| CTA | T>A | 9.49 × 10^-4^ | 1.52 × 10^-4^ | 2.37 × 10^-3^ | 1.49 × 10^-4^ |
| CTC | T>A | 1.56 × 10^-3^ | 2.29 × 10^-4^ | 2.88 × 10^-3^ | 2.75 × 10^-4^ |
| CTG | T>A | 1.97 × 10^-3^ | 2.50 × 10^-4^ | 4.36 × 10^-3^ | 2.81 × 10^-4^ |
| CTT | T>A | 7.10 × 10^-4^ | 1.17 × 10^-4^ | 1.09 × 10^-3^ | 1.27 × 10^-4^ |
| GTA | T>A | 1.37 × 10^-3^ | 2.57 × 10^-4^ | 2.27 × 10^-3^ | 3.43 × 10^-4^ |
| GTC | T>A | 1.96 × 10^-3^ | 2.78 × 10^-4^ | 2.21 × 10^-3^ | 2.22 × 10^-4^ |
| GTG | T>A | 2.02 × 10^-3^ | 2.97 × 10^-4^ | 4.40 × 10^-3^ | 2.59 × 10^-4^ |
| GTT | T>A | 4.50 × 10^-4^ | 1.02 × 10^-4^ | 7.05 × 10^-4^ | 1.12 × 10^-4^ |
| TTA | T>A | 6.11 × 10^-4^ | 1.51 × 10^-4^ | 6.29 × 10^-4^ | 9.71 × 10^-5^ |
| TTC | T>A | 1.25 × 10^-3^ | 1.57 × 10^-4^ | 1.42 × 10^-3^ | 1.70 × 10^-4^ |
| TTG | T>A | 1.11 × 10^-3^ | 2.00 × 10^-4^ | 2.24 × 10^-3^ | 2.67 × 10^-4^ |
| TTT | T>A | 1.96 × 10^-4^ | 6.76 × 10^-5^ | 4.65 × 10^-4^ | 8.43 × 10^-5^ |
| ATA | T>C | 6.16 × 10^-4^ | 1.34 × 10^-4^ | 4.01 × 10^-4^ | 1.02 × 10^-4^ |
| ATC | T>C | 1.54 × 10^-4^ | 8.68 × 10^-5^ | 8.81 × 10^-5^ | 4.51 × 10^-5^ |
| ATG | T>C | 3.47 × 10^-4^ | 1.20 × 10^-4^ | 3.76 × 10^-4^ | 5.88 × 10^-5^ |
| ATT | T>C | 3.41 × 10^-4^ | 1.24 × 10^-4^ | 2.76 × 10^-4^ | 8.06 × 10^-5^ |
| CTA | T>C | 1.96 × 10^-4^ | 6.71 × 10^-5^ | 1.49 × 10^-4^ | 6.62 × 10^-5^ |
| CTC | T>C | 3.15 × 10^-4^ | 6.65 × 10^-5^ | 3.29 × 10^-4^ | 9.62 × 10^-5^ |
| CTG | T>C | 3.11 × 10^-4^ | 6.25 × 10^-5^ | 2.03 × 10^-4^ | 8.31 × 10^-5^ |
| CTT | T>C | 1.99 × 10^-4^ | 4.15 × 10^-5^ | 5.89 × 10^-4^ | 5.52 × 10^-5^ |
| GTA | T>C | 3.65 × 10^-4^ | 1.32 × 10^-4^ | 3.66 × 10^-4^ | 1.13 × 10^-4^ |
| GTC | T>C | 2.52 × 10^-4^ | 9.52 × 10^-5^ | 2.98 × 10^-4^ | 1.38 × 10^-4^ |
| GTG | T>C | 4.47 × 10^-4^ | 1.06 × 10^-4^ | 3.04 × 10^-4^ | 9.60 × 10^-5^ |
| GTT | T>C | 3.53 × 10^-4^ | 1.16 × 10^-4^ | 3.25 × 10^-4^ | 9.76 × 10^-5^ |
| TTA | T>C | 1.54 × 10^-4^ | 5.57 × 10^-5^ | 1.30 × 10^-4^ | 7.89 × 10^-5^ |
| TTC | T>C | 1.42 × 10^-4^ | 7.38 × 10^-5^ | 1.97 × 10^-4^ | 5.02 × 10^-5^ |
| TTG | T>C | 2.65 × 10^-4^ | 8.51 × 10^-5^ | 2.45 × 10^-4^ | 9.00 × 10^-5^ |
| TTT | T>C | 4.23 × 10^-4^ | 1.32 × 10^-4^ | 2.33 × 10^-4^ | 6.27 × 10^-5^ |
| ATA | T>G | 1.52 × 10^-4^ | 8.28 × 10^-5^ | 1.34 × 10^-4^ | 7.30 × 10^-5^ |
| ATC | T>G | 1.65 × 10^-4^ | 6.92 × 10^-5^ | 1.38 × 10^-4^ | 9.26 × 10^-5^ |
| ATG | T>G | 9.87 × 10^-5^ | 4.05 × 10^-5^ | 2.51 × 10^-4^ | 6.75 × 10^-5^ |
| ATT | T>G | 2.06 × 10^-4^ | 6.20 × 10^-5^ | 3.07 × 10^-4^ | 1.06 × 10^-4^ |
| CTA | T>G | 7.40 × 10^-5^ | 4.95 × 10^-5^ | 4.39 × 10^-4^ | 1.21 × 10^-4^ |
| CTC | T>G | 9.12 × 10^-5^ | 4.78 × 10^-5^ | 3.36 × 10^-4^ | 7.08 × 10^-5^ |
| CTG | T>G | 1.95 × 10^-4^ | 6.15 × 10^-5^ | 4.99 × 10^-4^ | 9.01 × 10^-5^ |
| CTT | T>G | 3.69 × 10^-4^ | 6.34 × 10^-5^ | 3.19 × 10^-4^ | 7.59 × 10^-5^ |
| GTA | T>G | 1.83 × 10^-4^ | 7.49 × 10^-5^ | 2.29 × 10^-4^ | 8.70 × 10^-5^ |
| GTC | T>G | 2.54 × 10^-4^ | 6.93 × 10^-5^ | 3.87 × 10^-4^ | 1.12 × 10^-4^ |
| GTG | T>G | 2.70 × 10^-4^ | 8.48 × 10^-5^ | 7.76 × 10^-4^ | 1.14 × 10^-4^ |
| GTT | T>G | 1.88 × 10^-4^ | 6.50 × 10^-5^ | 3.84 × 10^-4^ | 1.01 × 10^-4^ |
| TTA | T>G | 2.52 × 10^-4^ | 1.16 × 10^-4^ | 7.65 × 10^-5^ | 3.99 × 10^-5^ |
| TTC | T>G | 7.59 × 10^-5^ | 4.00 × 10^-5^ | 1.24 × 10^-4^ | 3.48 × 10^-5^ |
| TTG | T>G | 1.38 × 10^-4^ | 4.60 × 10^-5^ | 2.60 × 10^-4^ | 8.17 × 10^-5^ |
| TTT | T>G | 1.60 × 10^-4^ | 4.34 × 10^-5^ | 1.28 × 10^-4^ | 3.47 × 10^-5^ |

**Supplementary Table 19.** Distribution of mutations across genomic targets (spontaneous mutations).

| Target | WT | | *Neil1^-/-^* | |
| --- | --- | --- | --- | --- |
|  | Mean MF | SE | Mean MF | SE |
| chr1-1 | 6.77 × 10^-8^ | 5.48 × 10^-9^ | 3.93 × 10^-8^ | 7.70 × 10^-9^ |
| chr1-2 | 1.38 × 10^-7^ | 2.16 × 10^-8^ | 1.45 × 10^-7^ | 2.83 × 10^-8^ |
| chr2 | 2.71 × 10^-8^ | 9.64 × 10^-9^ | 4.51 × 10^-8^ | 2.34 × 10^-8^ |
| chr3 | 6.47 × 10^-8^ | 9.72 × 10^-9^ | 5.91 × 10^-8^ | 1.55 × 10^-8^ |
| chr4 | 5.85 × 10^-8^ | 6.66 × 10^-9^ | 7.66 × 10^-8^ | 6.31 × 10^-9^ |
| chr5 | 3.30 × 10^-8^ | 1.24 × 10^-8^ | 7.37 × 10^-8^ | 1.76 × 10^-8^ |
| chr6 | 2.71 × 10^-8^ | 9.72 × 10^-9^ | 5.74 × 10^-8^ | 1.55 × 10^-8^ |
| chr7 | 2.82 × 10^-8^ | 7.20 × 10^-9^ | 4.23 × 10^-8^ | 1.10 × 10^-8^ |
| chr8 | 9.11 × 10^-8^ | 1.89 × 10^-8^ | 8.41 × 10^-8^ | 1.97 × 10^-8^ |
| chr9 | 5.91 × 10^-8^ | 1.96 × 10^-8^ | 8.91 × 10^-8^ | 2.06 × 10^-8^ |
| chr10 | 4.02 × 10^-8^ | 9.26 × 10^-9^ | 5.04 × 10^-8^ | 1.18 × 10^-8^ |
| chr11 | 5.92 × 10^-8^ | 5.72 × 10^-9^ | 1.21 × 10^-7^ | 1.73 × 10^-8^ |
| chr12 | 5.97 × 10^-8^ | 1.59 × 10^-8^ | 7.87 × 10^-8^ | 2.91 × 10^-8^ |
| chr13 | 7.02 × 10^-8^ | 1.73 × 10^-8^ | 4.95 × 10^-8^ | 4.55 × 10^-9^ |
| chr14 | 8.99 × 10^-8^ | 2.18 × 10^-8^ | 1.07 × 10^-7^ | 1.77 × 10^-8^ |
| chr15 | 4.73 × 10^-8^ | 1.89 × 10^-8^ | 6.20 × 10^-8^ | 2.30 × 10^-8^ |
| chr16 | 3.52 × 10^-8^ | 9.07 × 10^-9^ | 5.87 × 10^-8^ | 7.07 × 10^-9^ |
| chr17 | 9.26 × 10^-8^ | 2.15 × 10^-8^ | 9.91 × 10^-8^ | 1.93 × 10^-8^ |
| chr18 | 7.14 × 10^-8^ | 1.71 × 10^-8^ | 6.57 × 10^-8^ | 1.94 × 10^-8^ |
| chr19 | 4.23 × 10^-8^ | 1.56 × 10^-8^ | 7.21 × 10^-8^ | 2.25 × 10^-8^ |

**Supplementary Table 20.** Distribution of mutations across genomic targets (AFB_1_-induced mutations).

| Target | WT | | *Neil1^-/-^* | |
| --- | --- | --- | --- | --- |
|  | Mean MF | SE | Mean MF | SE |
| chr1-1 | 1.41 × 10^-6^ | 8.67 × 10^-8^ | 1.78 × 10^-6^ | 7.19 × 10^-8^ |
| chr1-2 | 1.76 × 10^-6^ | 6.43 × 10^-8^ | 2.03 × 10^-6^ | 8.63 × 10^-8^ |
| chr2 | 1.35 × 10^-6^ | 6.63 × 10^-8^ | 1.76 × 10^-6^ | 1.07 × 10^-7^ |
| chr3 | 1.72 × 10^-6^ | 6.20 × 10^-8^ | 2.10 × 10^-6^ | 9.70 × 10^-8^ |
| chr4 | 1.55 × 10^-6^ | 8.04 × 10^-8^ | 1.74 × 10^-6^ | 9.18 × 10^-8^ |
| chr5 | 1.45 × 10^-6^ | 7.06E × 10^-8^ | 1.81 × 10^-6^ | 1.16 × 10^-7^ |
| chr6 | 7.01 × 10^-7^ | 3.72 × 10^-8^ | 8.36 × 10^-7^ | 2.47 × 10^-8^ |
| chr7 | 1.18 × 10^-6^ | 3.66 × 10^-8^ | 1.40 × 10^-6^ | 3.73 × 10^-8^ |
| chr8 | 1.60 × 10^-6^ | 9.44 × 10^-8^ | 1.97 × 10^-6^ | 7.39 × 10^-8^ |
| chr9 | 1.36 × 10^-6^ | 5.76 × 10^-8^ | 1.64 × 10^-6^ | 8.75 × 10^-8^ |
| chr10 | 6.37 × 10^-7^ | 4.52 × 10^-8^ | 5.67 × 10^-6^ | 2.22 × 10^-8^ |
| chr11 | 1.31 × 10^-6^ | 7.18 × 10^-8^ | 1.63 × 10^-6^ | 6.13 × 10^-8^ |
| chr12 | 1.40 × 10^-6^ | 4.82 × 10^-8^ | 1.57 × 10^-6^ | 5.16 × 10^-8^ |
| chr13 | 2.19 × 10^-7^ | 2.21 × 10^-8^ | 1.75 × 10^-7^ | 2.05 × 10^-8^ |
| chr14 | 1.97 × 10^-6^ | 1.46 × 10^-7^ | 2.30 × 10^-6^ | 1.08 × 10^-7^ |
| chr15 | 1.53 × 10^-6^ | 8.42 × 10^-8^ | 1.76 × 10^-6^ | 8.27 × 10^-8^ |
| chr16 | 1.66 × 10^-6^ | 9.24 × 10^-8^ | 2.07 × 10^-6^ | 1.11 × 10^-7^ |
| chr17 | 1.51 × 10^-6^ | 8.08 × 10^-8^ | 1.71 × 10^-6^ | 7.65 × 10^-8^ |
| chr18 | 1.54 × 10^-6^ | 9.70 × 10^-8^ | 1.94 × 10^-6^ | 1.26 × 10^-7^ |
| chr19 | 3.98 × 10^-7^ | 3.21 × 10^-8^ | 3.57 × 10^-7^ | 1.97 × 10^-8^ |

**Supplementary Table 21.** Mutation frequencies in nDNA targets grouped by location in either genic or intergenic region or by chromatin state.

| Experimental group | Genomic region | | Chromatin state | |
| --- | --- | --- | --- | --- |
|  | Genic,  MF, mean ± SE | Intergenic,  MF, mean ± SE | Euchromatin,  MF, mean ± SE | Heterochromatin,  MF, mean ± SE |
| WT, 6-mo,  Unexposed (n = 4) | (5.91 ± 0.63) × 10^-8^ | (6.03 ± 0.50) × 10^-8^ | (5.73 ± 0.72) × 10^-8^ | (6.44 ± 0.27) × 10^-8^ |
| *Neil1^-/-^*, 6-mo,  Unexposed (n = 4) | (7.25 ± 0.42) × 10^-8^ | (7.45 ± 0.20) × 10^-8^ | (6.50 ± 0.47) × 10^-8^ | (8.98 ± 1.03) × 10^-8^ |
| WT, 2.5-mo,  AFB_1_ exposed (n = 10) | (1.14 ± 0.04) × 10^-6^ | (1.44 ± 0.07) × 10^-6^ | (1.24 ± 0.05) × 10^-6^ | (1.40 ± 0.06) × 10^-6^ |
| *Neil1^-/-^*, 2.5-mo,  AFB_1_ exposed (n = 10) | (1.32 ± 0.05) × 10^-6^ | (1.74 ± 0.08) × 10^-6^ | (1.47 ± 0.06) × 10^-6^ | (1.67 ± 0.07) × 10^-6^ |

**Supplementary Table 22.** Tumors observed in livers of AFB_1_-exposed WT and *Neil1^-/-^* mice.

| Mouse ID | Genotype | Sex | Number of tumors | Tumor diameter, mm |
| --- | --- | --- | --- | --- |
| 414 | WT | male | 1 | 4 |
| 415 | WT | male | 1 | 3 |
| 423 | WT | male | 0 | N/A |
| 427 | WT | male | 2 | 1  1 |
| 428 | WT | male | 1 | 1.5 |
| 563 | *Neil1^-/-^* | male | 2 | 0.5  0.5 |
| 565 | *Neil1^-/-^* | male | 3 | 15  15  15 |
| 585 | *Neil1^-/-^* | male | 1 | 12.5 |
| 586 | *Neil1^-/-^* | male | 1 | 3 |
| 587 | *Neil1^-/-^* | male | 1 | 12.5 |
| 588 | *Neil1^-/-^* | male | 1 | 17.5 |
| 417 | WT | female | 0 | N/A |
| 424 | WT | female | 0 | N/A |
| 425 | WT | female | 0 | N/A |
| 431 | WT | female | 1 | 25 |
| 432 | WT | female | 1 | 20 |
| 460 | *Neil1^-/-^* | female | 0 | N/A |
| 461 | *Neil1^-/-^* | female | 1 | 10 |
| 465 | *Neil1^-/-^* | female | 0 | N/A |
| 466 | *Neil1^-/-^* | female | 0 | N/A |
| 467 | *Neil1^-/-^* | female | 0 | N/A |
| 468 | *Neil1^-/-^* | female | 0 | N/A |

**Supplementary Figures**

**
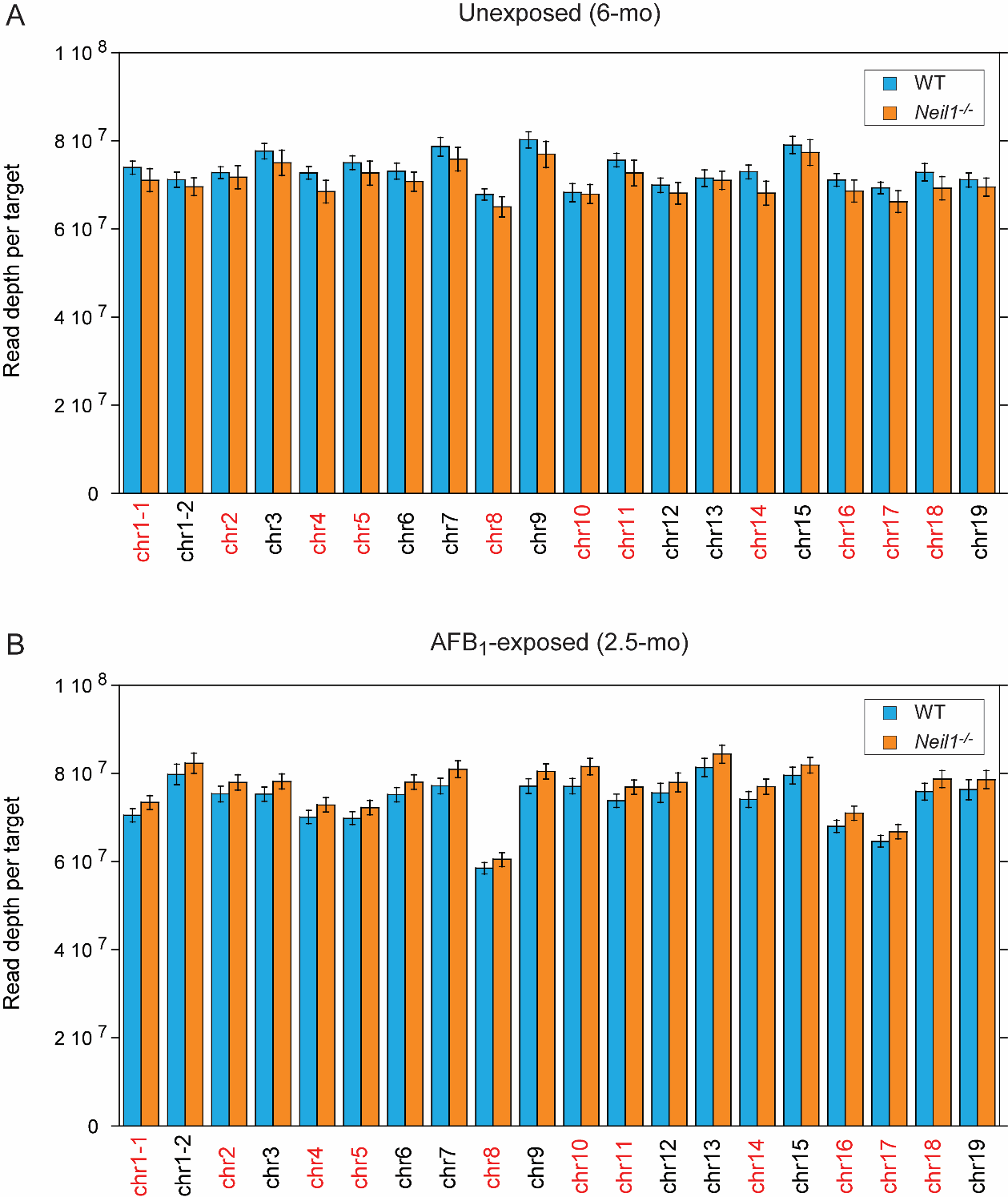
**

**Supplementary Figure 1**. Average DuplexSeq read depth per target for analyses of spontaneous (A) and AFB_1_-induced (B) mutations. Error bars represent standard errors. Intergenic targets are in red and genic targets are in black.


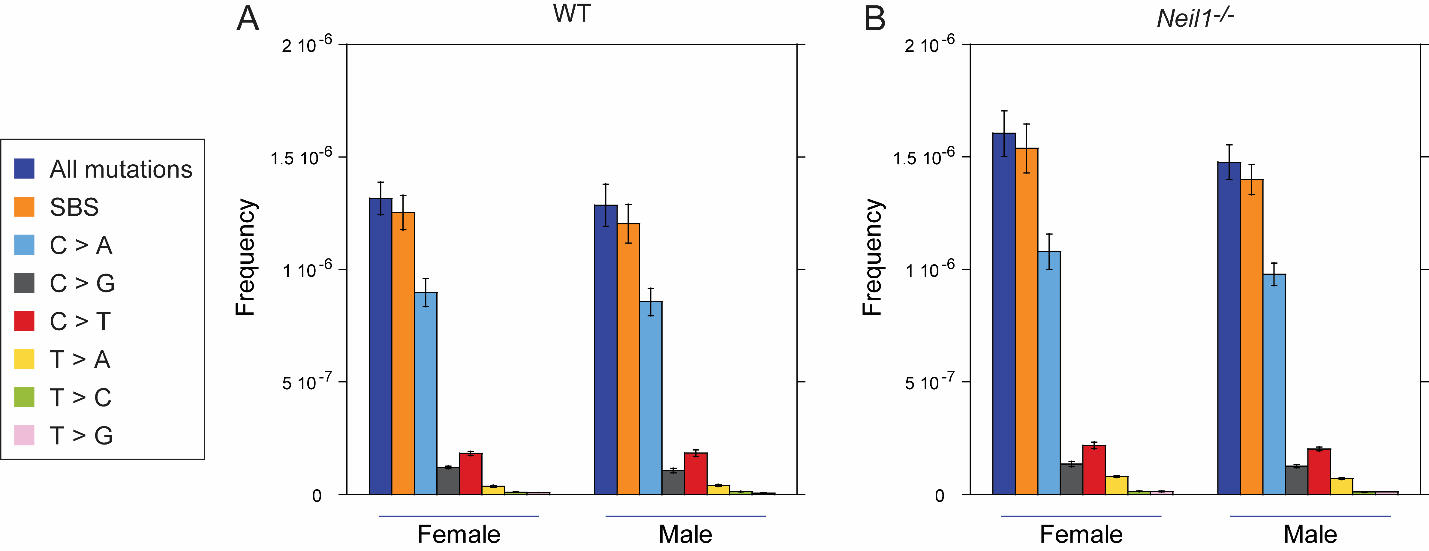


**Supplementary Figure 2**. Frequencies and spectra of mutations in nDNA of AFB_1_-exposed WT and *Neil1^-/-^* mice: female versus male (mean with standard errors).
